# Six Decades of Losses and Gains in Alpha Diversity of European Plant Communities

**DOI:** 10.1111/ele.70248

**Published:** 2025-11-02

**Authors:** Gabriele Midolo, Adam Thomas Clark, Milan Chytrý, Franz Essl, Stefan Dullinger, Ute Jandt, Helge Bruelheide, Olivier Argagnon, Idoia Biurrun, Alessandro Chiarucci, Renata Ćušterevska, Pieter De Frenne, Michele De Sanctis, Jürgen Dengler, Jan Divíšek, Tetiana Dziuba, Rasmus Ejrnæs, Emmanuel Garbolino, Estela Illa, Anke Jentsch, Borja Jiménez‐Alfaro, Jonathan Lenoir, Jesper Erenskjold Moeslund, Francesca Napoleone, Remigiusz Pielech, Sabine B. Rumpf, Irati Sanz‐Zubizarreta, Vasco Silva, Jens‐Christian Svenning, Grzegorz Swacha, Martin Večeřa, Denys Vynokurov, Petr Keil

**Affiliations:** ^1^ Department of Spatial Sciences, Faculty of Environmental Sciences Czech University of Life Sciences Prague Praha‐Suchdol Czech Republic; ^2^ Department of Biology University of Graz Graz Austria; ^3^ Department of Botany and Zoology, Faculty of Science Masaryk University Brno Czech Republic; ^4^ Division of BioInvasions, Global Change & Macroecology, Department of Botany and Biodiversity Research University of Vienna Vienna Austria; ^5^ Division of Biodiversity Dynamics and Conservation, Department of Botany and Biodiversity Research University of Vienna Vienna Austria; ^6^ Institute of Biology/Geobotany and Botanical Garden Martin Luther University Halle‐Wittenberg Halle Germany; ^7^ German Centre for Integrative Biodiversity Research (iDiv) Halle‐Jena‐Leipzig Leipzig Germany; ^8^ Conservatoire Botanique National Méditerranéen Hyères France; ^9^ Department of Plant Biology and Ecology, Faculty of Science and Technology University of the Basque Country UPV/EHU Bilbao Spain; ^10^ BIOME Lab, Department of Biological, Geological and Environmental Sciences Alma Mater Studiorum—University of Bologna Bologna Italy; ^11^ Faculty of Natural Sciences and Mathematics Ss. Cyril and Methodius University Skopje North Macedonia; ^12^ Forest & Nature Lab, Faculty of Bioscience Engineering Ghent University Gontrode Belgium; ^13^ Department of Environmental Biology Sapienza University of Rome Rome Italy; ^14^ Vegetation Ecology Research Group, Institute of Natural Resource Sciences (IUNR) Zurich University of Applied Sciences (ZHAW) Wädenswil Switzerland; ^15^ Bayreuth Center of Ecology and Environmental Research (BayCEER) University of Bayreuth Bayreuth Germany; ^16^ Department of Geobotany and Ecology, M.G. Kholodny Institute of Botany National Academy of Sciences of Ukraine Kyiv Ukraine; ^17^ Department of Ecoscience Aarhus University Aarhus Denmark; ^18^ ISIGE MINES Paris PSL Fontainebleau France; ^19^ Departament de Biologia Evolutiva, Ecologia i Ciències Ambientals, Facultat de Biologia Universitat de Barcelona Barcelona Spain; ^20^ Institut de Recerca de la Biodiversitat (IRBio) Universitat de Barcelona Barcelona Spain; ^21^ Disturbance Ecology and Vegetation Dynamics University of Bayreuth Bayreuth Germany; ^22^ Biodiversity Research Institute (IMIB) University of Oviedo‐CSIC‐Principality of Asturias Oviedo Spain; ^23^ UMR CNRS 7058 Ecologie et Dynamique des Systèmes Anthropisés (EDYSAN) Université de Picardie Jules Verne Amiens France; ^24^ Institute of Botany, Faculty of Biology Jagiellonian University Kraków Poland; ^25^ Department of Environmental Sciences University of Basel Basel Switzerland; ^26^ Egas Moniz Center for Interdisciplinary Research (CiiEM) Egas Moniz School of Health and Science Caparica Portugal; ^27^ Center for Ecological Dynamics in a Novel Biosphere (ECONOVO), Department of Biology Aarhus University Aarhus Denmark; ^28^ Botanical Garden University of Wrocław Wrocław Poland

**Keywords:** alpha diversity, autocorrelation, biogeographic regions, habitat specificity, latitudinal gradient, machine learning, nature conservation, random forests, statistical interpolation, vegetation resurvey

## Abstract

Biodiversity change forecasts rely on long‐term time series, but such data are often scarce in space and time. Here, we interpolated spatiotemporal changes in species richness using a new method based on machine learning that does not require temporal replication at sites. Using 698,692 one‐time sampled vegetation plots, we estimated trends in vascular plant alpha diversity across Europe and validated our approach against 22,852 independent time series. We found an overall near‐zero net change in species richness between 1960 and 2020. However, species richness generally declined from 1960 to 1980 and increased from 2000 to 2020 across habitats. Declines were most pronounced in forests, but trends varied across habitats and regions, with overall increases at higher latitudes and elevations, and declines or stable trends elsewhere. Our findings demonstrate how data without temporal replication can be used to reveal context‐dependent biodiversity dynamics, underscoring their importance for conservation and management.

## Graphical abstract

We developed a new method based on machine learning to interpolate spatiotemporal trends in vascular plant diversity across Europe from 1960 to 2020, utilising vegetation data without temporal replication. We found a near‐zero net change in species richness over the entire period, but declines from 1960 to 1980 were followed by increases from 2000 to 2020. These trends varied by habitat and region, highlighting the value of this new approach for understanding biodiversity change.

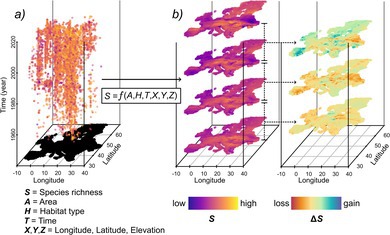

## 1 Introduction

Humans are driving major biodiversity changes worldwide (Díaz et al. 2019; IPBES 2019), but the direction and magnitude of these changes remain poorly understood across most taxa, regions, and scales (Gonzalez et al. 2016; Johnson et al. 2024). At the finest grain, that of the local biological community, losses and gains often offset each other (Bernhardt‐Römermann et al. 2015; Blowes et al. 2019; Dornelas et al. 2014; Jandt et al. 2022; Klinkovská et al. 2025; Pilotto et al. 2020; Vellend et al. 2013). In Europe, trends in plant diversity at the community level have been differentially impacted by various causes, such as agricultural intensification, biological invasions, climate change, conservation measures, and eutrophication (Finderup Nielsen et al. 2021; Gray et al. 2016; Steinbauer et al. 2018; Stevens et al. 2010; Vellend et al. 2017; Vilà et al. 2011). The relative strength of these drivers potentially varies over different historical periods (Klinkovská et al. 2024; Wesche et al. 2012), and their impacts may lag in time and only unfold after many decades (Dullinger et al. 2013). Consequently, the magnitude and direction of plant diversity change can differ across biogeographical regions and habitat types (Blowes et al. 2019; Pilotto et al. 2020). This wide variety of trends calls for time‐series analyses capable of identifying fine‐grain temporal changes in plant biodiversity across large spatiotemporal extents.

To quantify plant diversity trends, considerable efforts are underway to resurvey vegetation plots across Europe (Jandt et al. 2022; Klinkovská et al. 2025), which recently culminated in ReSurveyEurope (Knollová et al. 2024), a database collating hundreds of thousands of vegetation plot observations from numerous resurvey and monitoring projects. Despite the impressive collective effort and the extensive data now available, this database has significant geographical gaps, is biased toward well‐preserved habitats and sites, becomes increasingly sparse further back in time, and varies in the length of observation intervals between surveys—with a large fraction of vegetation plots resurveyed only once. As a result, we still have limited knowledge of how plant diversity at the community level (i.e., alpha diversity) has changed across various European biogeographical regions and habitats.

**FIGURE 1:**
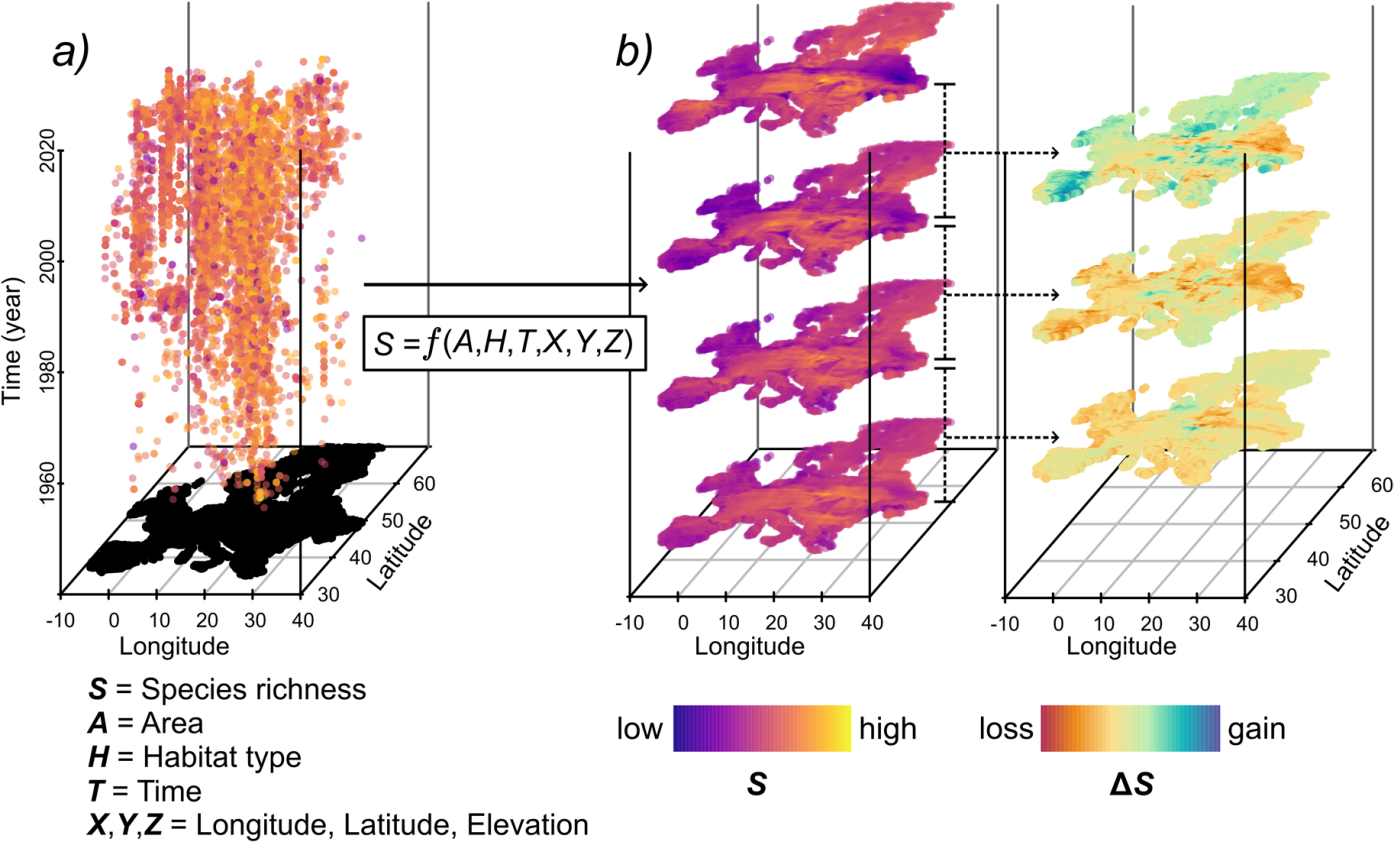
Workflow for spatiotemporal interpolation of plant species richness (*S*) and change in species richness (*ΔS*) in European vegetation plots. Species richness (*S*) from 698,692 vegetation plots that were sampled between 1945 and 2023 and not resurveyed in time, from the European Vegetation Archive (EVA) (Chytrý et al. 2016) and ReSurveyEurope (Knollová et al. 2024) is interpolated in each plot using machine learning as a function of plot area, time, space, habitat type, and their interactions, aiming to maximise prediction accuracy within the temporal dimension of the space–time cube (panel *a*). Interpolated values are then used to estimate temporal changes in species richness (panel *b*). Colour coding of the dots in panel (*a*) represents species richness recorded in 10,000 randomly selected plots from the EVA database for visualisation.

To address data gaps in space and time, we employ a novel approach (Keil and Chase 2022) that uses machine learning to interpolate temporal biodiversity change using only data that lack temporal replication at any given site. Our approach relies on the well‐established fact that biodiversity is spatially (Legendre 1993; Tobler 1970) and temporally (Dornelas et al. 2013) *autocorrelated*, which is caused by the continuity of species distributions across space and time because of dispersal limitation and environmental structure (Dornelas et al. 2013). Because of this autocorrelation, we propose that biodiversity can be interpolated jointly in space and time (i.e., in the *space–time cube*; Mahecha et al. 2020) (Figure 1). Specifically, we employed Random Forests (Breiman 2001; Wright and Ziegler 2017) to interpolate community‐level plant diversity in a multidimensional domain defined by geographical coordinates and time, while also accounting for the effect of varying plot area on species richness estimates (Dengler et al. 2020; Storch 2016). Our approach provides predictions of plant diversity within the temporal dimension, ultimately representing *interpolated time series* of biodiversity change.

To this end, we utilised 698,692 vegetation plots from the European Vegetation Archive (EVA) (Chytrý et al. 2016) and ReSurveyEurope (Knollová et al. 2024), sampled between 1945 and 2023 with plot sizes ranging from 1 to 1000 m^2^. Given its extensive temporal and geographic coverage, this dataset is well‐suited for interpolating biodiversity change. We modelled plot‐level alpha diversity (species richness) of vascular plants as a function of plot size, spatial coordinates (latitude, longitude, and elevation), sampling year, and habitat type (forest, grassland, scrub, or wetland). To validate our interpolation approach, we tested its ability to predict temporal species richness changes using 22,852 independent resurvey time series from ReSurveyEurope within the same period. Specifically, we trained the model on a single randomly selected observation from each time series and evaluated its predictions against observed species richness changes in ReSurveyEurope. Our model successfully predicted the direction of species richness changes and explained 41% of the variability in those changes (see Section 2.3). Having established the predictive power of our approach, we then applied it to temporally interpolate species richness dynamics across all vegetation plots sampled from 1960 to 2020, covering the last six decades across Europe.

## 2 Materials and Methods

### 2.1 Vegetation Plot Data

Our initial data set contained 1,679,403 vegetation‐plot observations available in the European Vegetation Archive (EVA) (Chytrý et al. 2016) and ReSurveyEurope (Knollová et al. 2024) (project no. 222; version 2024‐02‐06: https://doi.org/10.58060/jeht‐nr04). We restricted the analysis to plots with complete information on geographical location, habitat type, plot size, and sampling year, applying specific filters on the basis of each of these variables. We included only plots with coordinate uncertainty below 1 km and focused exclusively on habitats classified as forest, grassland, scrub, and wetland, following the EUNIS habitat classification system (Chytrý et al. 2020). Additionally, we only included plots within defined size ranges (1–100 m^2^ for non‐forest habitats, 100–1000 m^2^ for forests) and sampled between 1945 and 2023. Further details on data cleaning and preparation are listed in Appendix S1.

The application of these filters yielded a subset of 675,840 vegetation plot observations in EVA and 73,886 observations across 22,852 resurvey plots in ReSurveyEurope (Figure S4), which we used for all subsequent analyses. Both datasets were used for model training and interpolation. The ReSurveyEurope dataset was also used to test whether our approach could predict species richness dynamics observed in time series data (see Section 2.3). The final selection of plots included the following number of observations for each habitat type: 186,719 for forest (171,290 in EVA; 15,429 in ReSurveyEurope); 360,131 for grassland (309,286 in EVA; 50,845 in ReSurveyEurope); 70,334 for scrub (65,644 in EVA; 4690 in ReSurveyEurope); 132,542 for wetland (129,620 in EVA; 2922 in ReSurveyEurope). Across the selected time series in the ReSurveyEurope data, the median time span between the first and last observation was 15 years (ranging from 1 to 73 years; SD = 16 years).

### 2.2 Model Training

We used Random Forests (Breiman 2001) and Extreme Gradient Boosting (Chen and Guestrin 2016) to model plot‐level vascular plant species richness dependence on space (=elevation, latitude, and longitude) and time (=sampling year) while accounting for the effect of plot size and habitat type. We used these algorithms because they are better at modeling complex, non‐linear interactions between predictors, such as the species‐area relationship and its interaction with time (Keil and Chase 2022). This ability is crucial for maximizing the accuracy of our cross‐scale predictions of biodiversity metrics (such as species richness or occupancy) and for modeling interactions between temporal and spatial scales (Keil and Chase 2022, 2019; Leroy et al. 2024).

Our approach is based on *static* biodiversity data for spatiotemporal interpolation (Keil and Chase 2022). Throughout this analysis, we define *static data* as observations without temporal replication—specifically, vegetation plots that were surveyed only once, regardless of when or where they were sampled. We trained the final model used in the interpolation analyses presented in the results of this work on a total of 698,692 *static* vegetation plots, comprising 675,840 plots from EVA and 22,852 plots from ReSurveyEurope. Because plots from ReSurveyEurope have multiple observations, ranging from 2 to 46 (mean = 3.2; SD = 3.5), we randomly selected one observation from each plot. The remaining 51,034 plot observations from ReSurveyEurope were excluded from the final model fit but were later used for model evaluation as test data (see Section 2.3).

Modelling was fully conducted in R (version 4.4.2) (R Core Team 2024) with the *tidymodels* (Kuhn and Wickham 2020) package collection and included the main steps described below.

– *Training/Test set splitting*. We randomly split the dataset into training (80% of observations) and testing (20% of observations) datasets. We stratified the split by the response variable (=species richness) to balance its distribution in both data sets.
– *Model specification*. We fitted models using vegetation plots as observation units, with the following formula: *S*~*x* + *y* + *elevation* + *plot size* + *year + habitat type*; where *S* represents vascular plant species richness; *x* and *y* are the coordinates (in meters) of easting and northing, respectively; *elevation* corresponds to elevation above sea level (in meters); *plot size* is the area of the plot (in square meters); *year* is the year of sampling; and *habitat type* is a categorical variable describing the general habitat classification (‘forest’, ‘grassland’, ‘scrub’, or ‘wetland’). We used the ‘ranger’ (Wright and Ziegler 2017) and ‘xgboost’ (Chen et al. 2024) engines available in the *parsnip* R package (Kuhn and Vaughan 2024) for Random Forests and Extreme Gradient Boosting algorithms, respectively.
– *Hyperparameter tuning*. We performed hyperparameter tuning using a single 10‐fold cross‐validation on the training data. We selected the best combinations of hyperparameters based on the lowest root mean square error (RMSE). For the Random Forests, we set the number of trees to 1000 and used a regular grid of 25 combinations of other hyperparameters, setting the minimum number of data points in a node for further splitting (=‘node size’) to 2, 5, 10, 15, and 20, and the number of randomly sampled predictors (=‘mtry’) from 2 to 6. For XGBoost, we tuned all possible hyperparameters (except for the number of trees, which was set to 1000) using default tuning parameters available in the *dials* R package (Kuhn and Frick 2024). We reduced the grid search for XGBoost by fitting 50 combinations of hyperparameters using a space filling design with Latin hypercube grids with the ‘grid_space_filling’ function of the *dials* package. We used the optimal hyperparameter settings determined from our Random Forests tuning (node size = 5 and mtry = 3; yielding RMSE = 7.1 and *R* ^2^ = 0.685, as shown in Figure S7) as the default for the final model fit and subsequent analyses.
– *Model evaluation*. We evaluated the models using a 10‐fold cross‐validation (repeated three times) on the training data and on a separate testing dataset. We preferred this approach over block cross‐validation (Roberts et al. 2017), as it is not appropriate for interpolation tasks where spatial or temporal coordinates are used as predictors, since blocking would artificially limit the model's ability to learn from continuous gradients of space and time. Although this was not within the scope of our approach, we tested the performance of our model when generalising to unseen areas of space and time using spatial, temporal, and spatiotemporal block cross‐validation. We found good accuracy even though blocking was performed on the predictors (geographic coordinates and sampling year), which inevitably reduces any model's performance (Table S1). For details on the effects of spatial and temporal distance between the test and training data, see Appendix S2. We used RMSE (assessing deviation from the 1:1 relationship between predicted and observed species richness) and *R* ^2^ (=the squared Pearson correlation coefficient between observed and predicted values) to assess model performance. The Random Forests algorithm (RMSE = 7.0, *R* ^2^ = 0.69) performed better than the XGBoost algorithm (RMSE = 8.1, *R* ^2^ = 0.58; Table S2). Therefore, in all subsequent analyses, we exclusively applied Random Forests. Finally, we tested that the distribution of model residuals did not exhibit geographical clusters by using correlograms with the ‘spline.correlogram’ function of the *ncf* R package (Bjornstad 2022) (Figure S8) and by plotting the distribution of plot‐level residuals calculated from the testing data (observed minus predicted species richness), averaged at a 50 km resolution (Figure S9).

We also estimated the proportion of prediction variability explained by interactions between each pair of predictors as well as the proportion of joint effect variability of pairwise interactions by calculating *H* ^2^ statistics (Friedman and Popescu 2008) with the ‘hstats’ function from the *hstats* R package (Mayer 2024) (Figure S10). We utilized the ‘partial_dep’ function from the same package to visualize partial dependence plots (Figure S11).

### 2.3 Model Validation

To assess the reliability of our approach in estimating species richness dynamics, we trained separate Random Forest models on three *static* datasets: (*A*) including ReSurveyEurope data only, (*B*) including EVA data only, and (*C*) including both ReSurveyEurope and EVA. To make the data in ReSurveyEurope ‘*static*’ (i.e., select a single plot observation at each vegetation plot site) for datasets *A* and *C*, we randomly selected one observation from each of the 22,852 resurvey plots. We split each of the three datasets, using 80% of the data to train the models. We then evaluated their model performance (RMSE and *R* ^2^) separately based on the following observations from the three independent testing datasets:

1. *Species richness of the testing data*. Here, formal model evaluation was performed on 20% of the data not used for model training during the data split.
2. *Species richness of data from ReSurveyEurope*. This included all plots from ReSurveyEurope that were not used for model training, that is, the remaining 51,034 independent plots neither used in training nor in testing.
3. *Species richness change in ReSurveyEurope*. This was assessed using the log‐response ratio (lnRR) of species richness between the initial and final plots within each resurvey time series. A positive correlation indicates that changes in species richness obtained from model predictions can capture observed changes in species richness.

We repeated the model training procedure 100 times for dataset *A*, each with a new random selection of training and testing data from each time series. The model evaluation metrics showed that predictions were robust to random selection of plot combinations within the time series when tested using the criteria outlined in points 1, 2, and 3 (see Table S3).

Overall, our test demonstrated the feasibility of predicting species richness dynamics using interpolations from static data: when interpolating over ReSurveyEurope data, predicted and observed changes were positively correlated (RMSE = 0.39; *R* ^2^ = 0.41; Pearson correlation = 0.64) (Figure S12; Table S3). Predictions overall captured the observed direction of change, but they tended to slightly underestimate the observed magnitude of change, resulting in more conservative estimates (Figure S13). However, models struggled to accurately predict species richness changes in new, independent data, i.e., when EVA‐only trained models were tested in ReSurveyEurope (RMSE = 0.50; *R* ^2^ = 0.06; correlation = 0.23; Figure S12). This was likely because of an uneven spatial distribution of ReSurveyEurope plots relative to EVA plots (see Figure S2; Appendix S2), an overall higher error of predicting richness over two time periods for lnRR calculation, and a different temporal distribution of EVA plots located close to ReSurveyEurope plots. Finally, we found no significant differences in model validation results when comparing permanent and quasi‐permanent ReSurveyEurope plots (Figure S14); thus our approach is potentially robust against biases related to plot relocation.

In summary, our approach can explain up to 41% of the variability in species richness change over long temporal spans (Figure S3; more than 70 years, from 1945 to 2023). The evaluation of the model trained exclusively on EVA and tested on ReSurveyEurope data suggests that the results should not be geographically extrapolated beyond plot‐level predictions. For these reasons, we used the interpolated spatiotemporal model to predict solely along the temporal dimension (i.e., we did not project the models outside the spatial scope of our data) and restricted the predictions of species richness dynamics exclusively to the plots utilised in the main model, combining data from both EVA and ReSurveyEurope (see Section 2.2). We further examined the effects of spatial and temporal distance from the training data (see details in Appendix S2).

### 2.4 Interpolation of Species Richness Change

We used our model, trained on EVA and ReSurveyEurope data (see Section 2.2), to predict species richness for each year from 1960 to 2020. Predictions were made for the 660,748 plots included in the analysis and sampled during this period, while keeping other predictors fixed. To account for differences in plot size, we used the median plot size for each habitat type (forests: 300 m^2^, grasslands: 20 m^2^, scrub: 64 m^2^, wetlands: 50 m^2^) in our species richness predictions. To explore changes in species richness across the entire study period (1960–2020), we calculated the percentage change in interpolated species richness between 1960 and 2020 for each plot, as follows: $S_{\text{change}}=\frac{100*\left(S_{2020}-S_{1960}\right)}{S_{1960}}$. Similarly, we also calculated 21‐year changes observed in 1980, 2000, and 2020, relative to those in the year 1960, 1980, and 2000, respectively.

We also examined temporal trends in mean species richness across all plots within each habitat type and across plots located in seven European biogeographical regions. For each year, we calculated the estimated mean species richness across all plots per habitat type or biogeographic region, respectively, and used it as the response variable in relation to year in a linear regression, effectively estimating the mean change in the number of species per year. When plotting the relationship between time and species richness, we visualised both the ‘*confidence interval*’ around the mean as well as the ‘*prediction interval*’. These metrics were estimated using raw predictions from each individual tree trained in the Random Forests (*n* = 1000). We averaged the predictions across all plots at each time point for every tree. The *confidence interval* around the mean was calculated as the mean of the tree predictions (=mean ± 1.96 × SD/√*n*). The *prediction interval* was derived as the 0.05 and 0.95 quantiles of the distribution of mean predictions across all trees, and it provides the range of the predicted values for each year. We grouped predictions into biogeographical regions sourced from the data of the European Environment Agency (2016). We merged the arctic, boreal, and Scandinavian alpine regions into a single biogeographical unit to distinguish these regions from the other alpine regions with nemoral‐continental (e.g., the Alps, Carpathians, and Pyrenees) and nemoral‐submediterranean (e.g., Dinaric Alps and Rhodopes) vegetation (Preislerová et al. 2024).

To visualise the geographic distribution of species richness change across Europe, we aggregated plot‐level percentages of species richness change into 50 km × 50 km grids by averaging the predicted values of all plots in each raster cell. We plotted multiple maps for different periods (1960–1980, 1980–2000, 2000–2020, and 1960–2020) and habitat types. We report the standard deviation of predicted values in each cell in Figure S15. Furthermore, to account for the different temporal coverage in some cells (Figures S5 and S6), we calculated and mapped species richness change for each time period, including only plots sampled within each respective period (Figure S16). Similarly, we evaluated different metrics of species richness change, namely, log‐response ratios (Figure S17), the raw number of species lost or gained (Figure S18), and linear slope estimates (Figure S19) over three periods (1960–1980, 1980–2000, and 2000–2020) separately, and across the entire study period (1960–2020). Linear slope estimates were obtained by fitting linear regressions of predicted species richness against year for each plot, estimating the average number of species gained or lost per year over the assessed period. All these metrics quantifying diversity change exhibited overall consistent patterns with one another. We developed a Shiny web application (Chang et al. 2023) that allows users to interactively explore geographic patterns of species richness change in different habitats at higher resolution (10 km × 10 km) (available at: https://gmidolo.shinyapps.io/interpolated_s_change_app/).

## 3 Results and Discussion

### 3.1 Balanced Diversity Changes, but Shifting Dynamics: Early Losses, Late Gains

**FIGURE 2:**
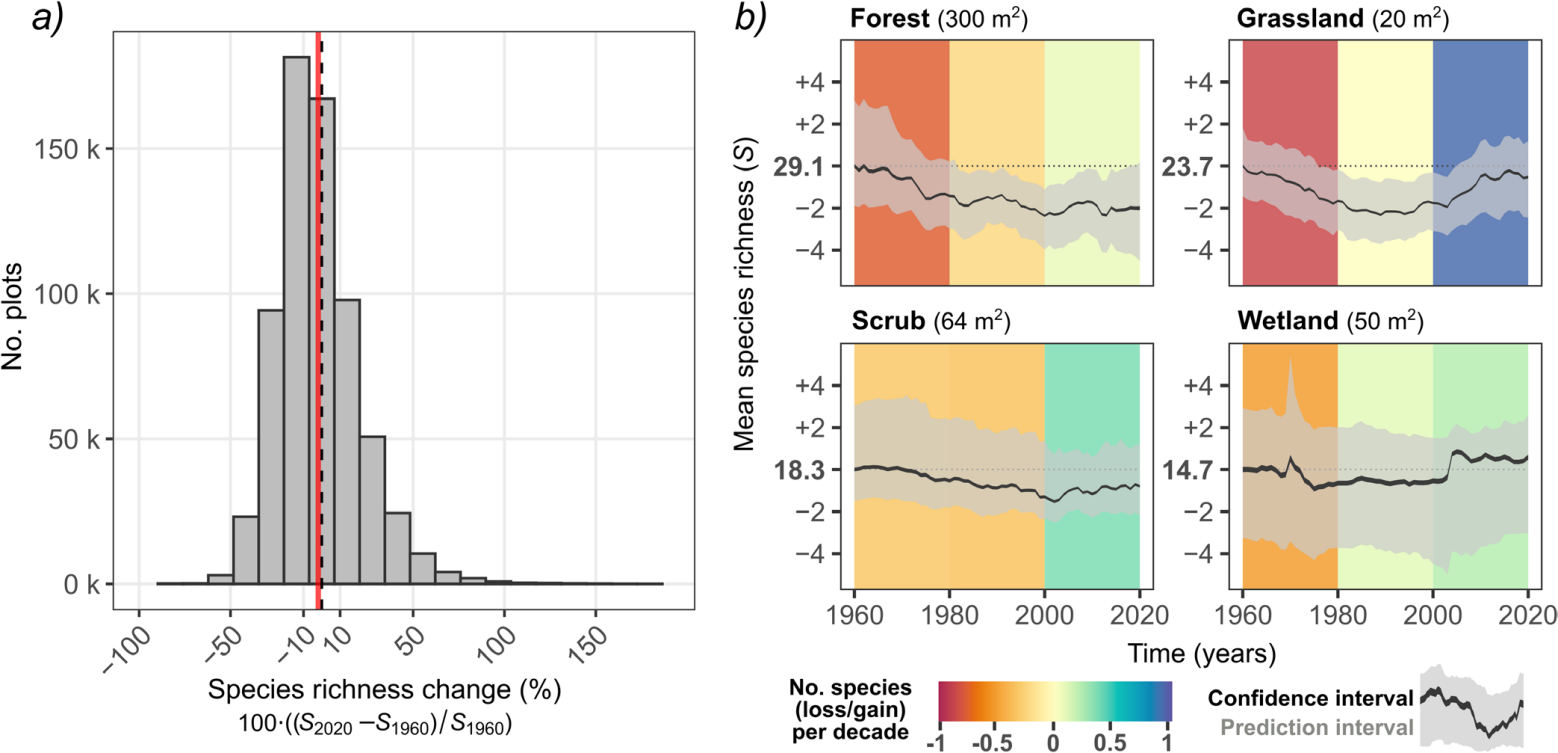
Summary of plant species richness changes over 61 years (1960–2020). Panel (*a*) shows the distribution of proportional change in interpolated plant species richness for the year 2020 compared to 1960 across 660,748 European vegetation plots sampled over that period. Dashed vertical line corresponds to 0% change; red solid line to the mean (=−1.9%). Panel (*b*) shows temporal trends in average species richness for each habitat type, calculated as the mean species richness across plots per year for each of the 1000 trees in the Random Forest. The trend across trees is summarised as the 95% confidence interval of the mean (black ribbon) and the prediction interval (grey ribbon). Linear regression slope estimates (coloured backgrounds) are fitted to species richness for three time periods: 1960–1980, 1980–2000, and 2000–2020. The *y*‐axis scale is standardised to the baseline mean species richness estimated for the year 1960. To standardise differences in plot size, species richness was predicted using a fixed plot size equal to the median plot size for each habitat (noted at the top of each panel).

We estimated close to zero mean net change in species richness (−2%) between 1960 and 2020 when averaged across all plots and habitat types (Figure 2a). This finding is similar to the results of previous large‐scale analyses of alpha diversity change, which show low net richness change across multiple time series (Bernhardt‐Römermann et al. 2015; Blowes et al. 2019; Dornelas et al. 2014; Jandt et al. 2022; Vellend et al. 2013). Although the overall species loss in our analysis was minor, this average trend hides a substantial proportion of plots exhibiting large changes over the past six decades: 15% of plots showed a steep decline, with losses of more than 20% of their initial species richness, whereas 19% of plots showed the opposite trend, with species gains of more than 20%.

**FIGURE 3:**
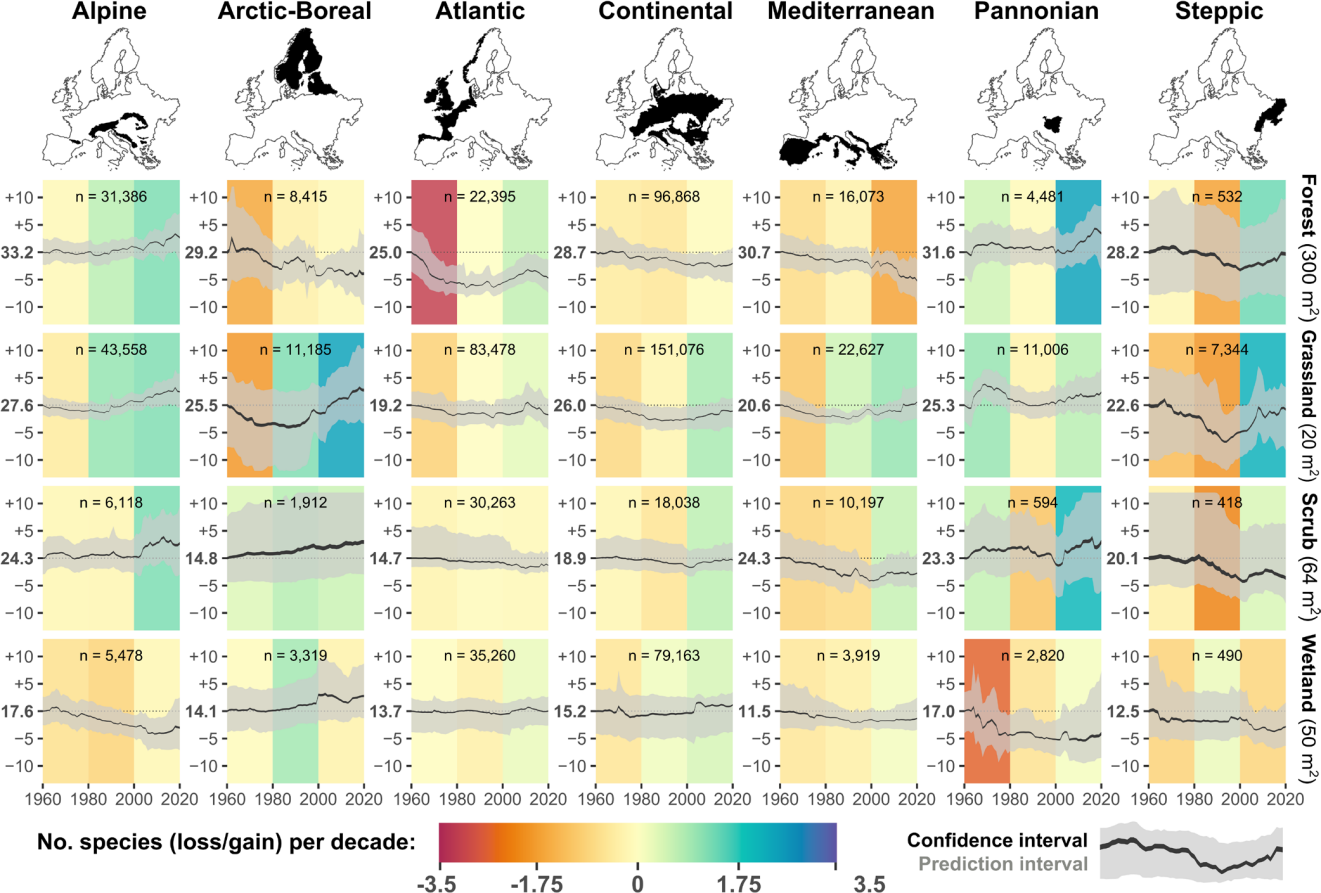
Trends of species richness change over 61 years (1960–2020) in seven European biogeographic regions. Each panel displays the estimated trend in mean interpolated species richness (*y*‐axis), calculated across all plots within a given biogeographic region and habitat type, per year, for each of the 1000 trees in the Random Forest. The trend across trees is summarised as the 95% confidence interval of mean (black ribbon) and the prediction interval (grey ribbon). The number of plots (*n*) is indicated at the top of each panel. Linear regression slope estimates (coloured backgrounds) are fitted to species richness for three time periods: 1960–1980, 1980–2000, and 2000–2020. The *y*‐axis scale is standardised to the baseline mean species richness estimated for the year 1960. Species richness was predicted using a fixed plot size equal to the median plot size for each habitat (noted at the right for each habitat).

We identified shifting dynamics over time characterised by a prevailing decline in species richness from 1960 to 1980 (continuing up to the 2000s in forests and scrubs), followed by gains in species richness from 2000 to 2020 across all habitat types (Figure 2b) and in most biogeographic regions (Figure 3). Although our approach cannot establish a causal link between species richness changes and potential underlying drivers, the greater losses detected during earlier decades align with well‐documented factors contributing to European biodiversity decline. These factors include agricultural intensification and eutrophication driven by nitrogen (N) and phosphorus (P) enrichment, along with acid deposition, all of which began increasing in the first half of the 20th century and peaked in its second half (Araújo et al. 2008; Fuchs et al. 2015; Schöpp et al. 2003; de Vries et al. 2024). Each of these drivers has likely impacted different habitats to a different extent, such as acidic deposition in forests (Hédl 2004), and soil drainage and nitrogen deposition favouring encroachment by generalist species in wetlands (Sperle and Bruelheide 2021) and grasslands (Stevens et al. 2010). As recently shown for the flora of the Czech Republic (Klinkovská et al. 2024), industrialization and land‐use intensification during the 1960–1980s have generally advantaged species adapted to anthropogenic disturbances and high nutrient availability. Especially high rates of eutrophication in European countries during this period likely contributed to species richness declines by promoting the dominance of a few species favoured by high nitrogen availability (Staude et al. 2020; Stevens et al. 2010). These declines were lessened over time when species numbers gradually reduced as compositions shifted to more nitrophilous vegetation (Bobbink et al. 2010), with possible species richness recovery following reduced N input (Storkey et al. 2015).

**FIGURE 4:**
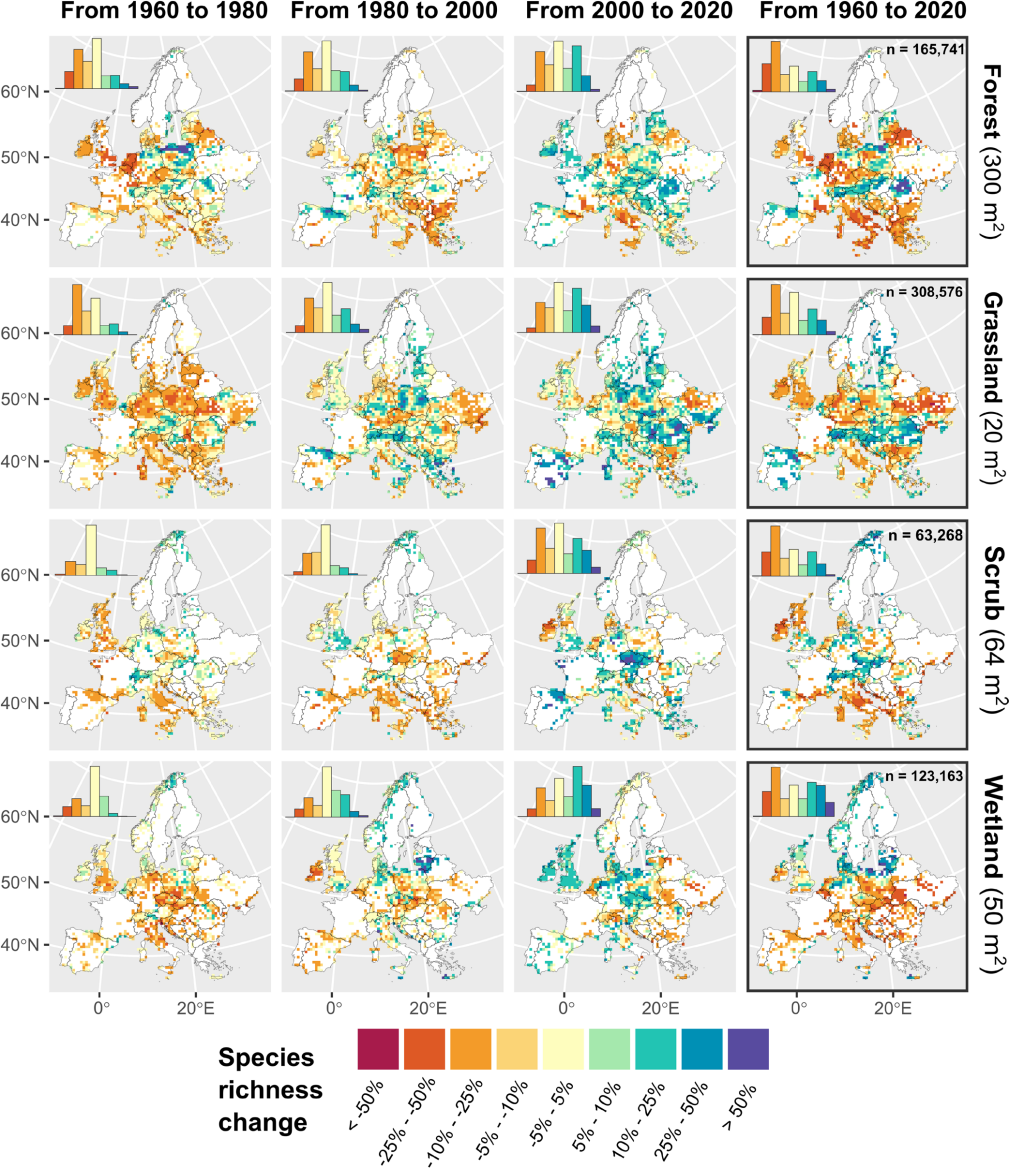
Geographic patterns of plant species richness change in Europe. The maps show the mean percentage change in plot‐level species richness between two time points, aggregated within 50 km × 50 km grid cells. Only grid cells containing at least five plots sampled between 1960 and 2020 are shown. Histograms (upper left of each panel) illustrate the distribution of percentage change classes across all plots. The number of plots (*n*) for each habitat type is shown in the panels on the right, which estimate richness changes from 1960 to 2020. Species richness was predicted using a fixed plot size equal to the median plot size for each habitat type (noted on the right for each habitat type). An interactive dashboard plotting species richness changes at a finer resolution is available at https://gmidolo.shinyapps.io/interpolated_s_change_app/.

Subsequently, the increased occupancy of warm‐adapted and non‐native species (Klinkovská et al. 2024), along with overall range shifts of species tracking their thermal niches in response to climate change (Rumpf et al. 2018), may have contributed to local species enrichment in recent decades (2000–2020). This trend was particularly noticeable at higher latitudes (e.g., in open habitats of the boreal region) and in mountainous areas (Figures 3 and 4), which corroborates previous studies focusing on these ecosystems and regions (Steinbauer et al. 2018; Thuiller et al. 2005). Furthermore, species richness gains observed since the 2000s could partially reflect the Europe‐wide abatement of airborne anthropogenic nitrogen and sulphur deposition from the 1990s onwards (Sutton et al. 2011; de Vries et al. 2024), as well as more recent improvements in nature conservation and restoration policies supported by the European Union (e.g., the 1992 Habitats Directive; European Economic Committee 1992). Preferential sampling could partly explain the observed positive trends in species richness in some regions over the last decades. However, to our knowledge, this bias applies only to those regions where older vegetation surveys, focusing on phytosociological classification (sometimes omitting certain species from the plots, leading to an underestimation of richness), shifted toward sampling of better‐preserved vegetation over more recent years (e.g., because of monitoring of protected areas). Yet, older surveys often targeted floristically richer sites, whereas recent efforts more often include degraded sites too, potentially balancing these trends.

### 3.2 Habitat‐ and Region‐Specific Diversity Change

We found highly context‐dependent trajectories of species richness change (Figures 3 and 4), consistent with studies challenging the notion of unidirectional biodiversity change (Dornelas et al. 2023; Johnson et al. 2024; Pilotto et al. 2020) and supporting the idea that biodiversity change depends on habitat type (Klinkovská et al. 2025), geographic location (Bernhardt‐Römermann et al. 2015; Blowes et al. 2019), and, most crucially, the time period considered. Although significant geographic heterogeneity in interpolated species richness trends was observed across Europe, a few distinct geographic patterns emerged. Overall, colder regions, namely the alpine and the arctic‐boreal zones, showed increases in species richness, although localized declines were observed in wetlands and forests within these regions; conversely, other regions displayed either stable trends or overall losses.

When comparing 2020 to 1960, forest habitats showed the highest proportion of plots with substantial declines of species richness compared to other habitats, with 25% of plots estimated to have lost ≤ 20% of species, and a mean change of −6%. Large declines in forests are interpretable as results of alteration of management practices (i.e., cessation of coppicing and forest grazing coupled with increased canopy density), resulting in a shift towards species‐poorer communities representative of denser, moister, and nutrient‐richer conditions (i.e., mesification) (Hédl et al. 2010; Lelli et al. 2021). Our findings partially contrast with the synthesis by Bernhardt‐Römermann et al. (2015) of resurvey studies on forest vegetation in Europe, which reported more balanced trends for this habitat type. However, their study focused on temperate forests only and covered fewer old observations, potentially reducing their chance to detect losses that occurred in the 1960s or earlier (particularly in the Atlantic region). Indeed, we identified constant declines of species richness in boreal and Mediterranean forests (Figure 3). Interestingly, forests in the Alpine region displayed an opposite, positive trend. These variations across biogeographic regions reflect the notion that local and regional drivers specific to forest habitats—such as changes in large ungulate densities, management practices, and their interactions with nitrogen deposition and global warming—create context‐dependent impacts on plant species richness dynamics of forest vegetation (Bernhardt‐Römermann et al. 2015; Perring et al. 2018; Staude et al. 2020).

Grasslands displayed more balanced trends, with a net species richness change close to zero (mean: −1.5%) over the whole study period. The high heterogeneity of grassland diversity trends across Europe (Figure 4) could reflect, among many other drivers, highly localized management practices, ranging from strong intensification to complete abandonment, creating a patchy mosaic of biodiversity trends across European grasslands (Shipley et al. 2024). Conversely, wetlands had the most polarised results, with large fractions of plots showing high gains and losses (≥ 20%) in 25% and 18% of the plots, respectively, resulting in a slightly positive average change (mean: +4%) across the whole study period. Compared to other habitats, wetlands also displayed a more distinct geographical gradient: plots with increasing richness or no change were primarily located at higher latitudes (above 55° N). Given the substantial pressures affecting most European wetland ecosystems (Verhoeven 2014), we did not expect large gains in wetlands. This sharp latitudinal pattern may reflect changes in local hydrological regimes (e.g., soil drainage) that promote the encroachment of generalist vascular plant species, resulting in higher species richness found in northern European mires (Kolari et al. 2021; Pedrotti et al. 2014). The higher magnitude of change in wetlands also likely reflects their generally lower baseline vascular species richness (see Figure S7), where the addition of a few species can strongly affect proportional gains in species richness. Nonetheless, most wetlands at lower latitudes, especially in Central Europe (Figure 4), experienced large losses in species richness. This finding accords with previous studies of wetlands in central and southern European lowlands, which have documented climate change toward warmer conditions, combined with drastic increases in human water extraction from natural systems, as key drivers of wetland specialist losses (Navrátilová et al. 2022; Sperle and Bruelheide 2021).

## 4 Advances, Limitations, and Implications for Addressing Biodiversity Change

With this study, we provide the first continental‐scale analysis of local biodiversity change over continuous time and space, unlocking an unprecedented amount of vegetation‐plot data collected over more than six decades. Compared to previous large‐scale analyses of time series data (Bernhardt‐Römermann et al. 2015; Blowes et al. 2019; Dornelas et al. 2014; Jandt et al. 2022; Klinkovská et al. 2025; Pilotto et al. 2020; Vellend et al. 2013), our approach enabled us to (i) unravel these trends across a broad geographic extent and different habitat types and (ii) compare them to common baselines from the same time period. This was previously difficult to achieve at the continental scale because of data limitations and because past methods rarely utilised static data to inform analyses of biodiversity dynamics (Jandt et al. 2011). Our method addresses the latter and can potentially be applied to other taxonomic groups and spatial grains (Keil and Chase 2022; Leroy et al. 2024) to robustly forecast biodiversity change even in regions lacking dedicated time series data. Additionally, our method can be applied to individual species occurrences, offering potential for studies mapping single‐species dynamics and composition changes, although this may involve considerable computational costs. Our work is intended to complement, rather than replace, the efforts of field ecologists to resurvey vegetation and collect new data, which are the fundamental source for assessing spatial and temporal patterns of biodiversity. Indeed, geographically and temporally representative long‐term monitoring of habitats across Europe is essential for effectively assessing, preserving, and restoring biodiversity (Moersberger et al. 2024). Such monitoring data are crucial not only for understanding biodiversity change but also for enhancing the validation of our method from non‐systematic data sources like those employed here.

Although our approach provides a robust assessment of regional‐scale biodiversity trends (i.e., the average local trend in a larger region), as indicated by the results of our model validation, it is likely weaker at making precise predictions at individual sites, as indicated by the 59% of variance in species richness change that was not explained by our predictions on time series data. In addition, although habitat changes (e.g., a transition from grassland to scrub) can be integrated into model predictions, our approach does not account for local land‐use changes or intensification, such as the destruction of surveyed plant communities because of deforestation, urbanisation, or agricultural conversion. In other words, our method is conservative in estimating local species richness change, assuming the preservation of each community type within the time window assessed. We suggest that future studies of systems where relevant proxies for biodiversity change drivers are known at the observation unit level could incorporate these as predictors, providing a way to test these drivers. Furthermore, we caution that this method may yield unreliable estimates in regions lacking sufficient temporal coverage of geographically close observations in areas that are poorly sampled over time (but see Appendix S2).

Species richness dynamics revealed by our study have several key implications for biodiversity assessment and conservation planning in Europe. Our results suggest that vegetation plots experiencing local losses in species richness could also experience gains in the future. Yet, in many cases, a local increase in species richness should not necessarily be interpreted as an improvement in conservation status but could instead indicate habitat quality deterioration (namely, losses in habitat specialists in favour of generalist or non‐native species colonising plant communities and increasing local alpha diversity) (Jandt et al. 2022; Klinkovská et al. 2025). Further investigation of long‐term plant diversity trends at broader scales and beyond alpha diversity (e.g., habitat specialist occupancy) is needed to confirm whether the recent rise in local species richness across habitats results from biotic homogenization by generalists and alien species at the expense of specialists. Given the large geographic heterogeneity of diversity trends that we uncovered, we nonetheless emphasise the importance of recognising regional variations when implementing conservation and restoration actions. This means tailoring the implementation of joint EU policies, such as the Agri‐Environment Schemes (European Commission 2017), the Common Agricultural Policy (CAP) (European Commission 2012), and the Nature Restoration Law (European Commission 2023), to prioritize local and habitat‐specific conservation and restoration needs.

## Author Contributions

G.M. and P.K. developed the original idea and conceptualised the methodology. G.M. led the investigation, data processing, visualisations, analysis, drafted the original manuscript, and led the writing. P.K. supervised the project and contributed to the original draft. P.K., A.T.C., and F.E. acquired funding. All authors except G.M., A.T.C., and P.K. contributed the data used in the analysis. All authors reviewed the manuscript or contributed data.

## Peer Review

The peer review history for this article is available at https://www.webofscience.com/api/gateway/wos/peer‐review/10.1111/ele.70248.

## Supporting information


**Data S1:** ele70248‐sup‐0001‐Supinfo.docx.

## Data Availability

The data and R code to fully reproduce the analyses are available in the GitHub repository: https://github.com/gmidolo/interpolated_S_change. Output files, including model fits, are available on Zenodo: https://doi.org/10.5281/zenodo.17357776. An interactive map exploring interpolated spatiotemporal changes in species richness can be accessed at: https://gmidolo.shinyapps.io/interpolated_s_change_app (GitHub repository: https://github.com/gmidolo/interpolated_S_change_app). The use of the data for additional publications and access to complete and original vegetation data is only possible through a request to the EVA Coordinating Board (see https://euroveg.org/eva‐database/).
