# Supplementary material for: Six Decades of Losses and Gains in Alpha Diversity of European Plant Communities
Source: Ecol Lett. 2025 Nov 2;28(11):e70248. doi: 10.1111/ele.70248 (PMC12579928; doi:10.1111/ele.70248)
Supplement: Supplementary file 1 — Data S1: ele70248‐sup‐0001‐Supinfo.docx. [file ELE-28-0-s001.docx]

Supporting Information

**Six Decades of Losses and Gains in Alpha Diversity of European Plant Communities**

*Ecology Letters*

<https://doi.org/10.1111/ele.70248>

**Authors**: Midolo, G., Clark, A. T., Chytrý, M., Essl, F., Dullinger, S., Jandt, U., Bruelheide, H., Argagnon, O., Biurrun, I., Chiarucci, A., Ćušterevska, R., De Frenne, P., De Sanctis, M., Dengler, J., Divíšek, J., Dziuba, T., Ejrnæs, R., Garbolino, E., Illa, E., Jentsch, A., Jiménez-Alfaro, B., Lenoir, J., Moeslund, J. E., Napoleone, F., Pielech, R., Rumpf, S. B., Sanz-Zubizarreta, I., Silva, V., Svenning, J.-C., Swacha, G., Večeřa, M., Vynokurov, D., Keil, P.

**Appendix S1: Additional details on the preparation of vegetation data**

We calculated species richness (*S*) as the number of species in each plot, including subspecies and aggregates as individual species. We focused exclusively on vascular plants and excluded non-vascular plant taxa from the analysis because non-vascular plants were often not recorded. Species nomenclature was based on Euro+Med Plant-Base (Euro+Med PlantBase 2024) and that of aggregates following the EUNIS-ESy system (Chytrý et al. 2020). When counting species richness, we also included taxa identified only at the genus level. Before calculating species richness, we merged species occurrences within the same plot observation if a species was recorded in multiple vegetation layers. We checked for duplicates and removed any identical plot observations (based on plot size, year of sampling, geographic coordinates, and species composition) from the European Vegetation Archive (EVA) that were also present in ReSurveyEurope, despite having different plot ID numbers. Similarly, we also checked each dataset internally to ensure there were no duplicate records within EVA and ReSurveyEurope.

We restricted the analysis to plots with complete information on geographical location, habitat type, plot size, and sampling year, applying specific filters based on each of these variables, as described below.

- ***Geographic location.*** We included only plots located in Europe with available geographical coordinates. We excluded plots from Iceland, Svalbard, Russia, and Turkey. We transformed geographic coordinates to latitude and longitude in meters using ETRS89 / UTM zone 32N (EPSG:25832) projection (hereinafter, ‘northing’ and ‘easting’, respectively) and used this projection throughout the analysis. To complete the information on the spatial location of all plots included in the analyses, we extracted elevation at plot location using a Digital Elevation Model with 90-m horizontal resolution from the European Space Agency (European Space Agency 2024). For the ReSurveyEurope data, we included both permanent plots (83% of the final dataset), which were resurveyed at precisely relocated sites, and quasi-permanent plots (17%), which lacked accurate relocation information. The coordinates of repeated observations for the same plot in ReSurveyEurope are not always consistent, likely due to variations in plot relocation error. Thus, to minimize potential bias in species richness interpolation during model validation, we excluded any plots in ReSurveyEurope where at least one observation was located more than 100 meters from other observations at that same plot site. This procedure resulted in the removal of 3,655 observations from 582 plots. Additionally, all experimentally manipulated permanent plots were excluded from the ReSurveyEurope dataset.
- ***Habitat types.*** We classified the vegetation plots into habitat types based on species composition and cover using the European Nature Information System (EUNIS) Habitat Classification expert system (Chytrý et al. 2020) (version 2021-06-01). We grouped plots into level-1 EUNIS habitat types and restricted the analyses only to plots categorized either as forest (code ‘T’), grassland (code ‘R’), scrub (code ‘S’), or wetland (code ‘Q’). We also included coastal dunes and sandy shore habitats (code ‘N1’) and classified them either as forest, grassland, or scrub depending on the physiognomy of their level-3 EUNIS habitat. We discarded all plots that were not categorized in one of the habitats above, such as man-made vegetation and marine habitats. Data reported in the Danish Nature Database (Nielsen et al. 2012) (which lacks species-cover data needed for classification into EUNIS habitats by the expert system) were classified into level-1 EUNIS category using the Annex I habitat conversion sheet of the same database (European Environment Agency 2023).
- ***Plot size.*** We included only plots with sizes ranging from 1 to 100 m^2^ for grasslands, scrub and wetlands, and from 100 to 1000 m^2^ for forests to exclude outliers as well as potential mistakes of plot size reported in the datasets of EVA and ReSurveyEurope (Midolo et al. 2024). We excluded all plots with no information on plot size.
- ***Sampling year***. We excluded plots sampled before 1945 due to a much lower number of plots compared to later periods. For model training, testing, and evaluation, we focused on plots sampled between 1945 and 2023. However, to further mitigate potential biases associated with older sampling protocols and overall fewer plots before 1960, or due to reporting lags for studies that were conducted within the last few years, we restricted interpolations to plots sampled from 1960 to 2020 (see ‘Interpolation of diversity change’ section). The temporal distribution of ReSurveyEurope data matched that of the EVA data (Figure S4), and we had broad temporal coverage of plots sampled in most European regions (Figure S5-S6).

**References**

Chytrý, M., Tichý, L., Hennekens, S.M., Knollová, I., Janssen, J.A.M., Rodwell, J.S., et al. (2020). EUNIS Habitat Classification: Expert system, characteristic species combinations and distribution maps of European habitats. *Appl Veg Sci*, *23*, 648–675.

Euro+Med PlantBase. (2024). Euro+Med PlantBase – the information resource for Euro-Mediterranean plant diversity. Available at: <https://www.europlusmed.org>. Last accessed 22 November 2024.

European Environment Agency. (2023). EUNIS terrestrial habitat classification 2021_1 with crosswalks to Annex I in separate rows. 2023.

European Space Agency. (2024). Copernicus GLO-90 Digital Elevation Model. Distributed by OpenTopography. Available at: <https://doi.org/10.5069/G9028PQB>. Last accessed 12 August 2024.

Midolo, G., Axmanová, I., Divíšek, J., Dřevojan, P., Lososová, Z., Večeřa, M., et al. (2024). Diversity and distribution of Raunkiær’s life forms in European vegetation. Journal of Vegetation Science, 35.

Nielsen, K., Bak, J., Bruus, M., Damgaard, C., Ejrnæs, R., Fredshavn, J., et al. (2012). NATURDATA.DK – Danish monitoring program of vegetation and chemical plant and soil data from non-forested terrestrial habitat types. *Biodiversity & Ecology, 4*, 375–375.

**Appendix S2: Spatial and temporal distance effects on model performance**

***Spatial distance effects.*** To evaluate how spatial distance influences the prediction error of our main model, we selected 131,581 testing data points sampled between 1961 and 2020, grouping them into six 10-year intervals to control for temporal distance. For each decade, we computed the minimum distance from each testing point to the nearest training point sampled within the same decade. Distances were calculated using a 1-km resolution raster representing proximity to the training data for each respective decade. Testing data were then grouped by distance classes, and the root mean squared error (RMSE) was calculated for each distance class and decade to assess how prediction error changes with increasing spatial distance.


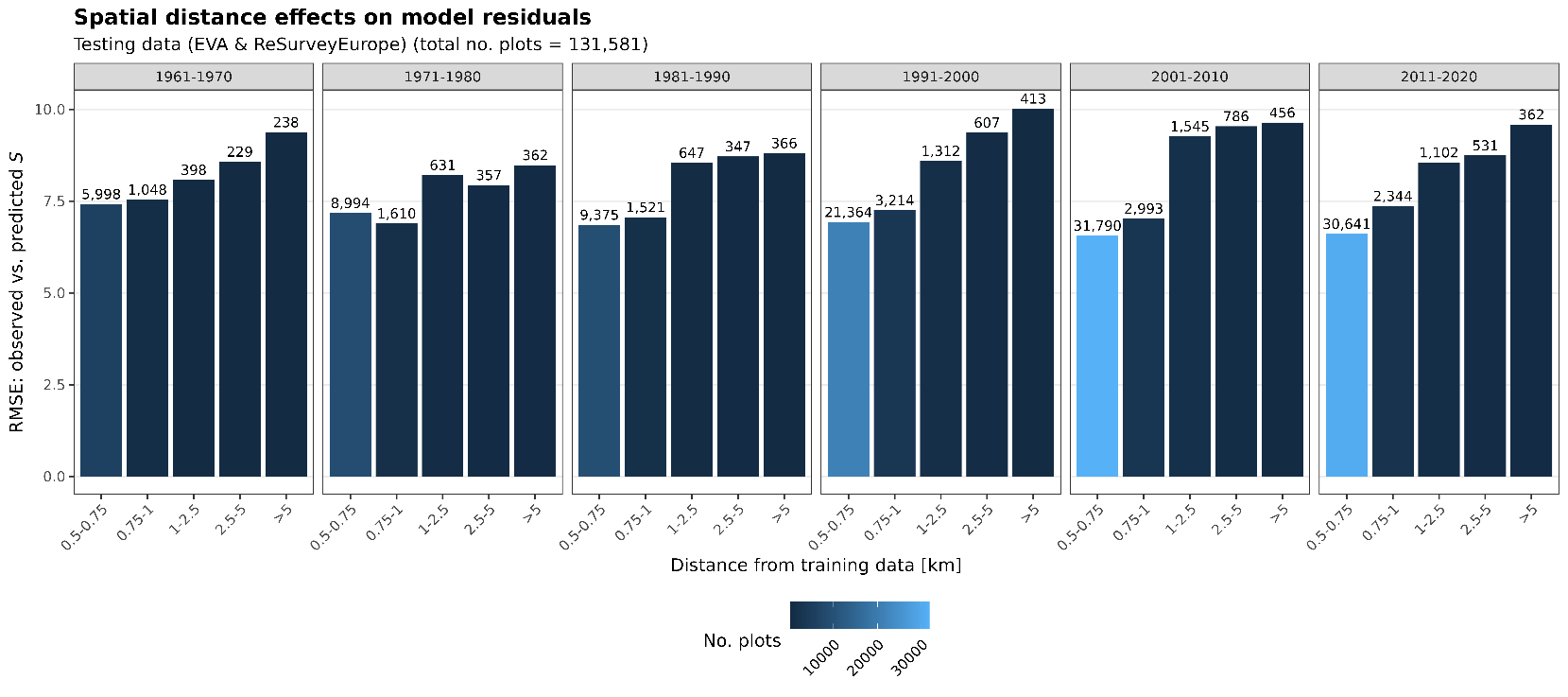
As expected, model performance moderately decreased with increasing spatial distance, particularly for testing points located more than 1 km from the nearest training observation (Figure S1). This trend was consistent across all decades. These results help explain the performance drop observed when models trained on EVA data are tested on spatially distinct locations from ReSurveyEurope (see Figure S12), as ReSurveyEurope plots are placed at greater distances than training data in EVA (Figure S2).

**Figure S1**: Model evaluation scores (root mean squared error, RMSE) for the Random Forest model, with RMSE scores of the testing data grouped by minimum distance classes from the training data within each time period.


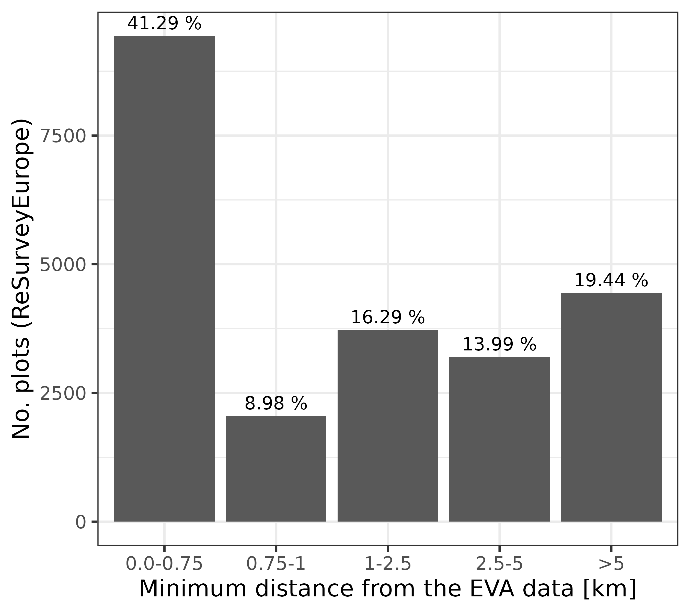


**Figure S2**: Distance of ReSurveyEurope plots from the EVA data. Plot count (and total percentages) are counted for each distance classes (same used in Figure S1). About 50% of ReSurveyEurope plots are located > 1 km distance from EVA plots.

***Temporal distance effects.*** We also assessed how temporal distance from the training data affects model accuracy using time-series data from ReSurveyEurope (see ‘Model Validation’). The testing dataset included 22,852 observed changes in species richness between the start and end of each time series, spanning 1945 to 2023 (see Figure S12). A new model was trained using a random subset of observations drawn from either the start or end of each time series, and its performance was evaluated by comparing predicted versus observed changes in species richness (log-response ratios) across testing data grouped by five-year temporal distance.
Overall, the model performed well in interpolating temporal change (Figure S3). Although prediction error was lower for shorter time spans (1–5 years), we did not observe a consistent increase in error with longer temporal distances. This suggests that the model is capable of capturing biodiversity trends over the time window assessed, provided there is sufficient spatial information to support temporal inference.

**
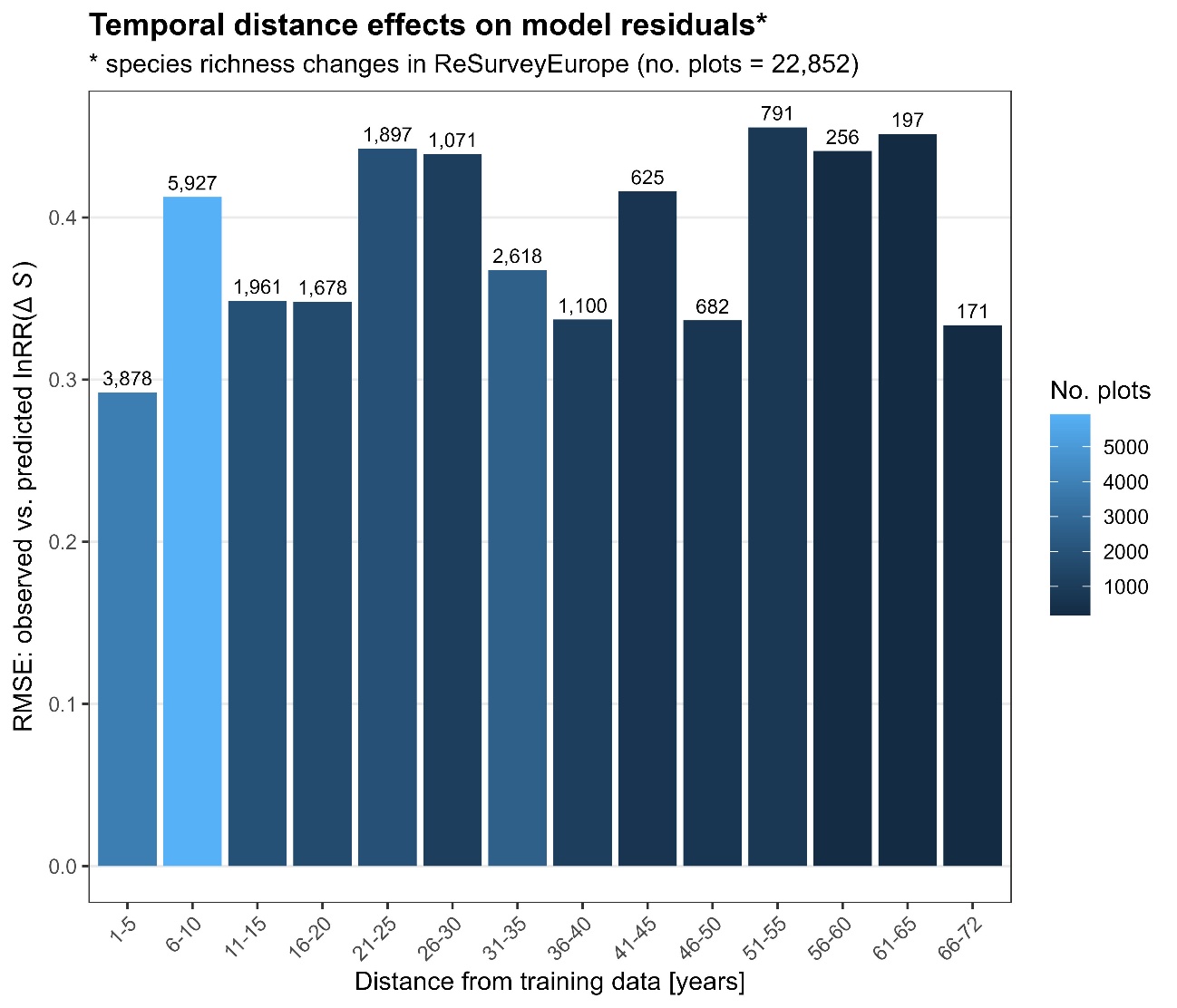
**

**Figure S3**: Model evaluation scores (root mean squared error, RMSE) for the Random Forest model trained without temporal replication and tested on time series data. The RMSE values are grouped by temporal distance classes from the training data.


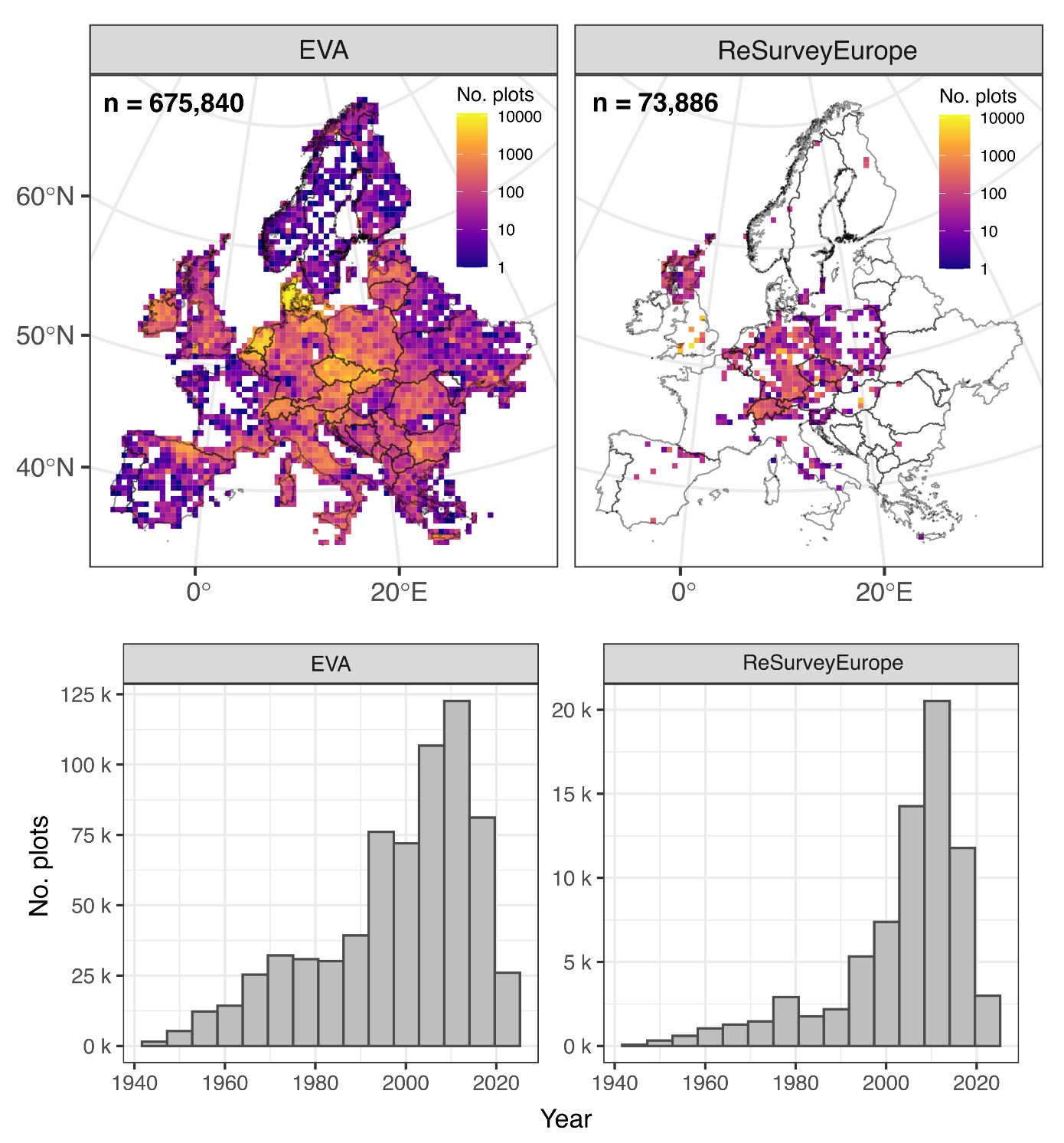


**Figure S4**: Spatial (top panels; 50 × 50 km resolution) and temporal (bottom panels) distribution across plot observations of the European Vegetation Archive (EVA) and ReSurveyEurope.


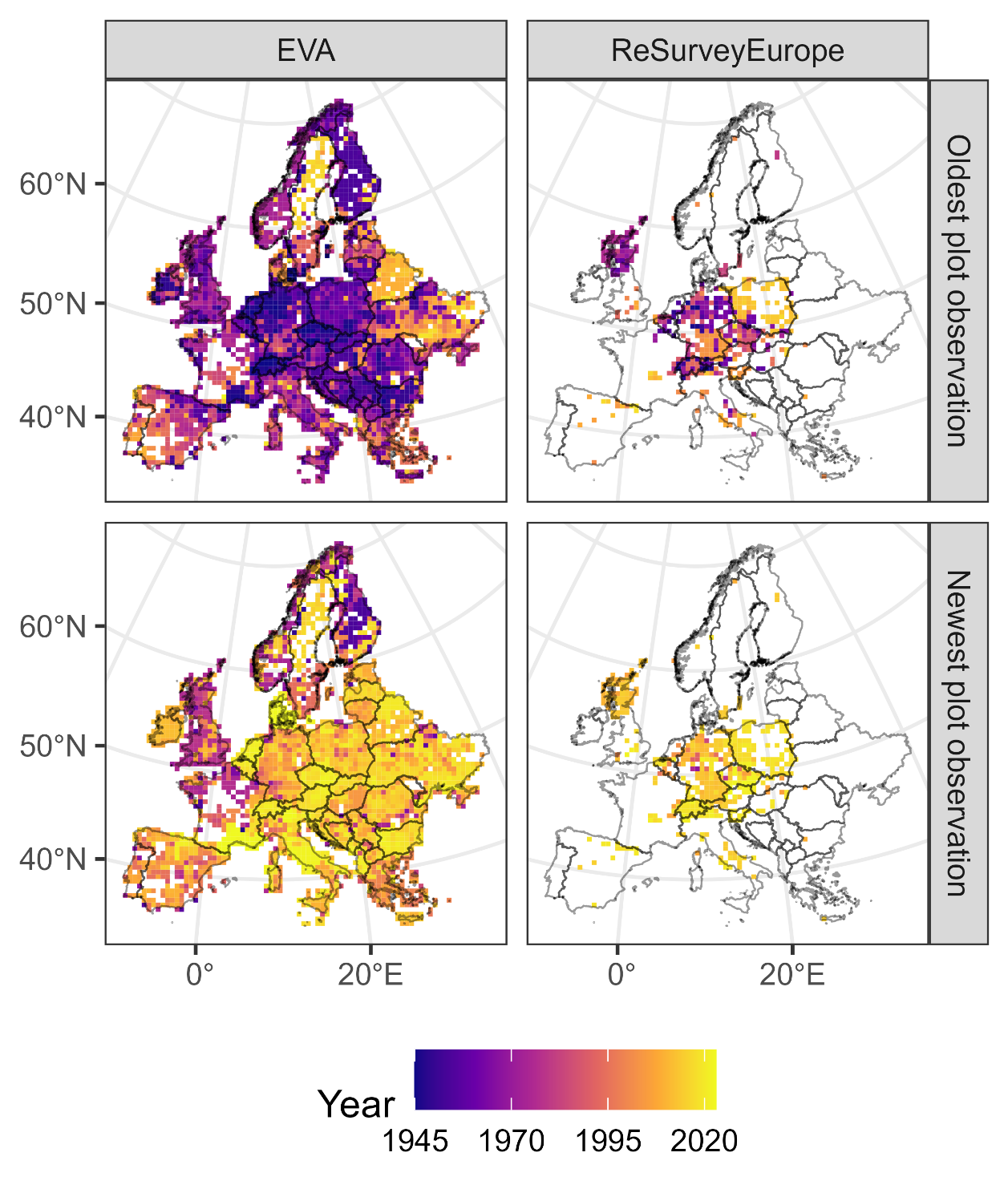
**Figure S5**: Year of the oldest (top panels) and newest (lowest panels) plot observation located in each grid cell (50 × 50 km resolution) across the vegetation plots of the European Vegetation Archive (EVA) and ReSurveyEurope.


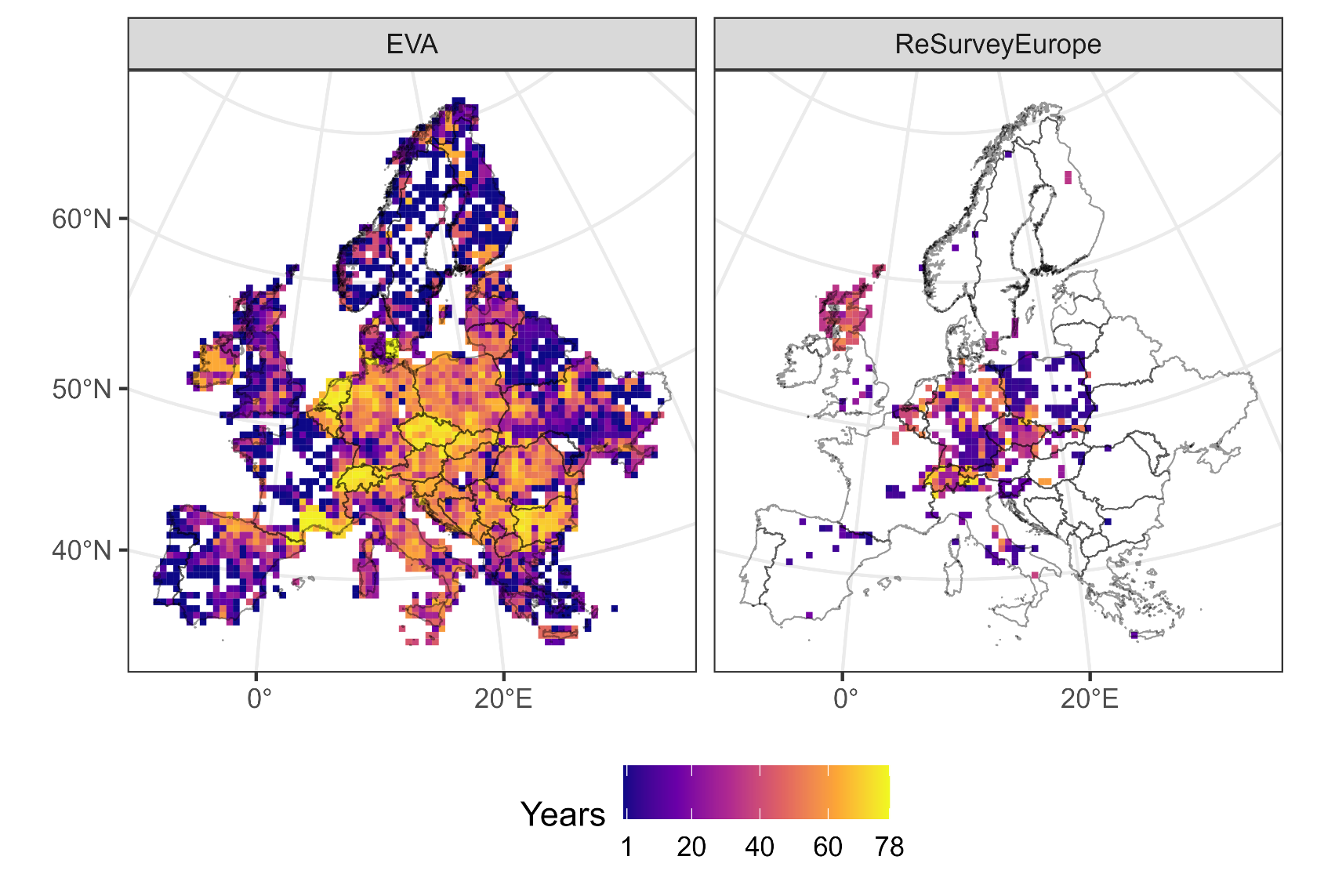
**Figure S6**: Temporal span (difference in years between the newest and the oldest plot observation) located in each cell on a 50 × 50 km resolution grid across the vegetation plots of the European Vegetation Archive (EVA) and ReSurveyEurope.

**Table S1:** Mean and standard deviation (SD) of evaluation metrics (root mean square error, *RMSE*; coefficient of determination using squared Pearson correlation of observed vs. predicted, *R^2^*) of the Random Forests model tested on *Spatial*, *Temporal*, and *Spatio-Temporal* block cross-validation (CV). To evaluate model’s ability to generalize (extrapolate) in space and time, observations were grouped using three distinct blocking strategies: 1) in the *Spatial block CV* we evaluated the model’s performance on unseen geographic areas by grouping plot observations at three different grid resolutions (1 km, 10 km, and 100 km) and performed a 10-fold CV for each resolution separately (i.e., using 10% of randomly selected blocks for testing, remaining 90% for training at each fold); 2) in the *Temporal block CV* we assessed the model’s ability to predict values for different time periods by setting aside plot observations from distinct 10-year periods (from 1961 to 2020) for testing (thus using 6-fold CV); 3) in the *Spatio-Temporal block CV* we used k-means clustering (with 100 clusters) to group plot observations based on their proximity in space and time, and then performed a 10-fold CV.

| **Blocking strategy** | | **No. of partitions (folds)** | **mean *RMSE* (SD)** | **mean *R*^2^ (SD)** |
| --- | --- | --- | --- | --- |
| 1) *Spatial block CV* | 1 km | 10 | 8.29 (0.091) | 0.566 (0.010) |
|  | 10 km | 10 | 8.97 (0.162) | 0.491 (0.019) |
|  | 100 km | 10 | 9.96 (0.536) | 0.369 (0.055) |
| 2) *Temporal block CV* | | 6 | 9.87 (0.454) | 0.399 (0.037) |
| 3) *Spatio-Temporal block CV* | | 10 | 9.65 (0.432) | 0.408 (0.036) |

**Table S2**: Evaluation metrics (root mean square error, *RMSE*; coefficient of determination using squared Pearson correlation of observed vs. predicted, *R^2^*) of the tested algorithms (Random Forests and XGBoost) on the full dataset tested on 10-fold random cross-validation (CV) with tree repeats over the training dataset (558,952) and last fit on the testing dataset (139,740).

|  | **Random Forests** | | **XGBoost** | |
| --- | --- | --- | --- | --- |
|  | *RMSE* | *R^2^* | *RMSE* | *R^2^* |
| **Random CV on the training set** | 7.07 (SE: 0.0064) | 0.685 (SE: 0.0005) | 8.19 (SE: 0.0050) | 0.580 (SE: 0.0006) |
| **Last fit on the testing set** | 7.02 | 0.688 | 8.14 | 0.583 |


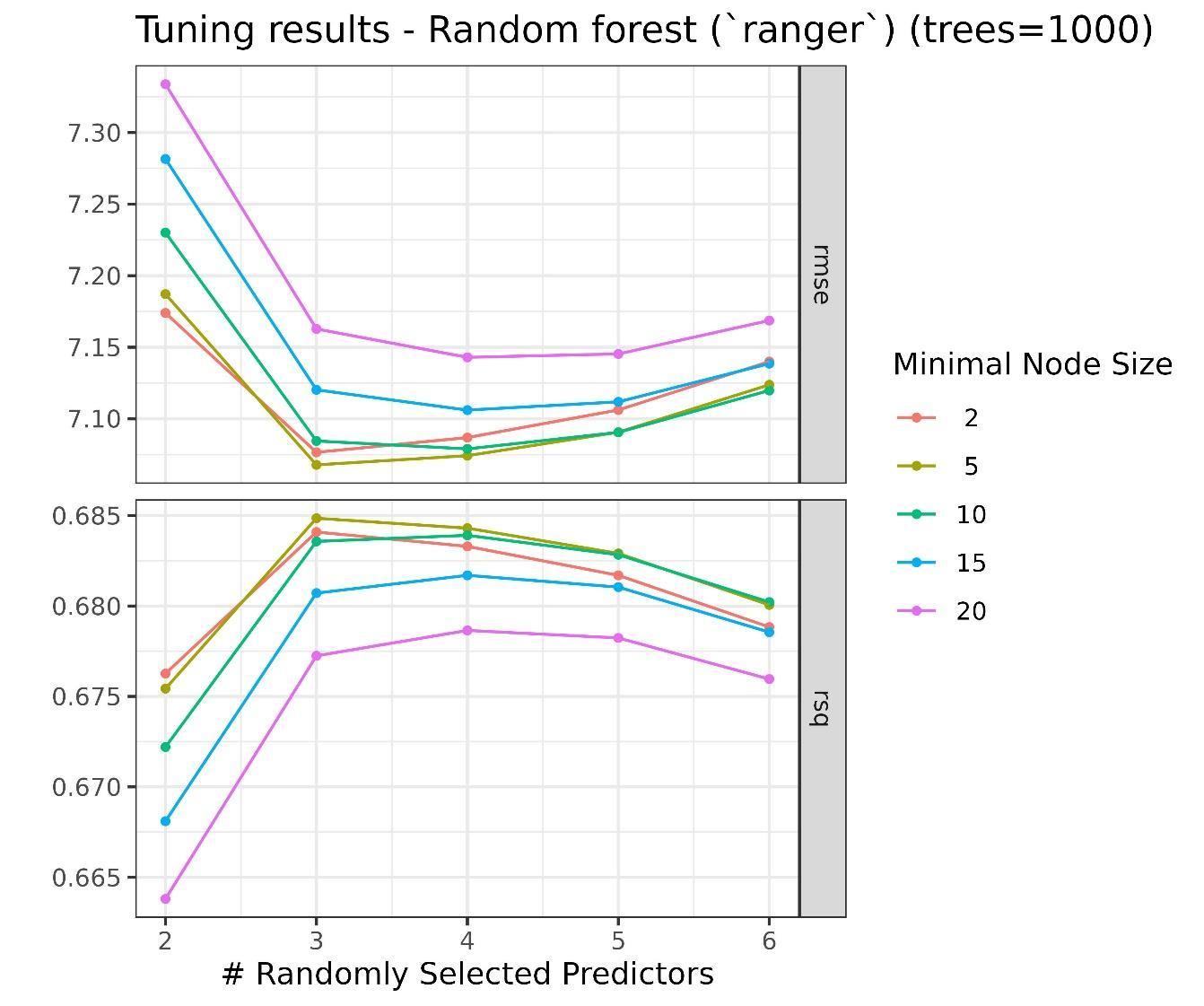
**Figure S7**: Hyperparameter tuning results of minimal node size and the number of randomly selected predictors in Random Forests.

**
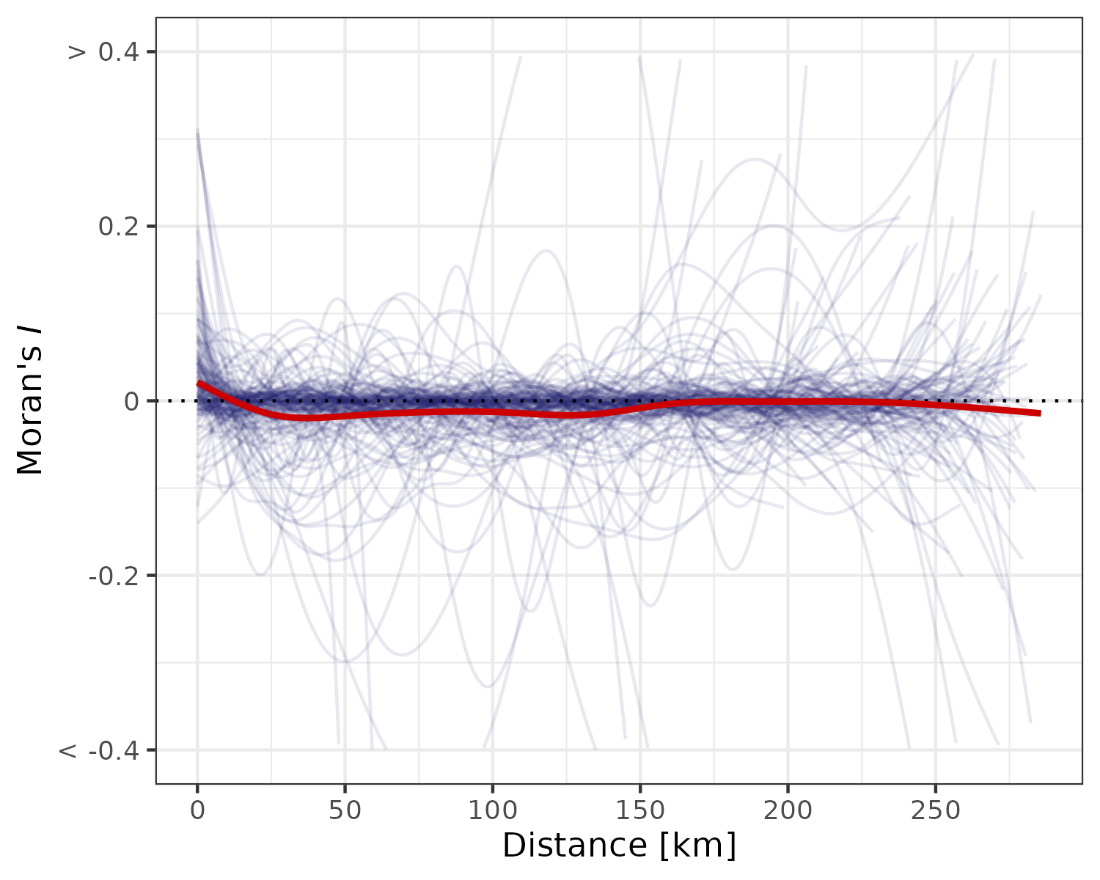
Figure S8**: Spatial autocorrelation of model residuals assessed using Moran’s *I*. Correlograms show the relationship between Moran’s *I* and spatial distance within hexagonal grid cells (250 km wide; ~144,337 m side length), each containing at least 30 testing plots. A loess smoother summarizes the overall pattern. As expected, residual spatial autocorrelation is low and centered around zero, indicating that incorporating geographic coordinates as predictors effectively accounted for spatial structure in the data.

**Figure S**
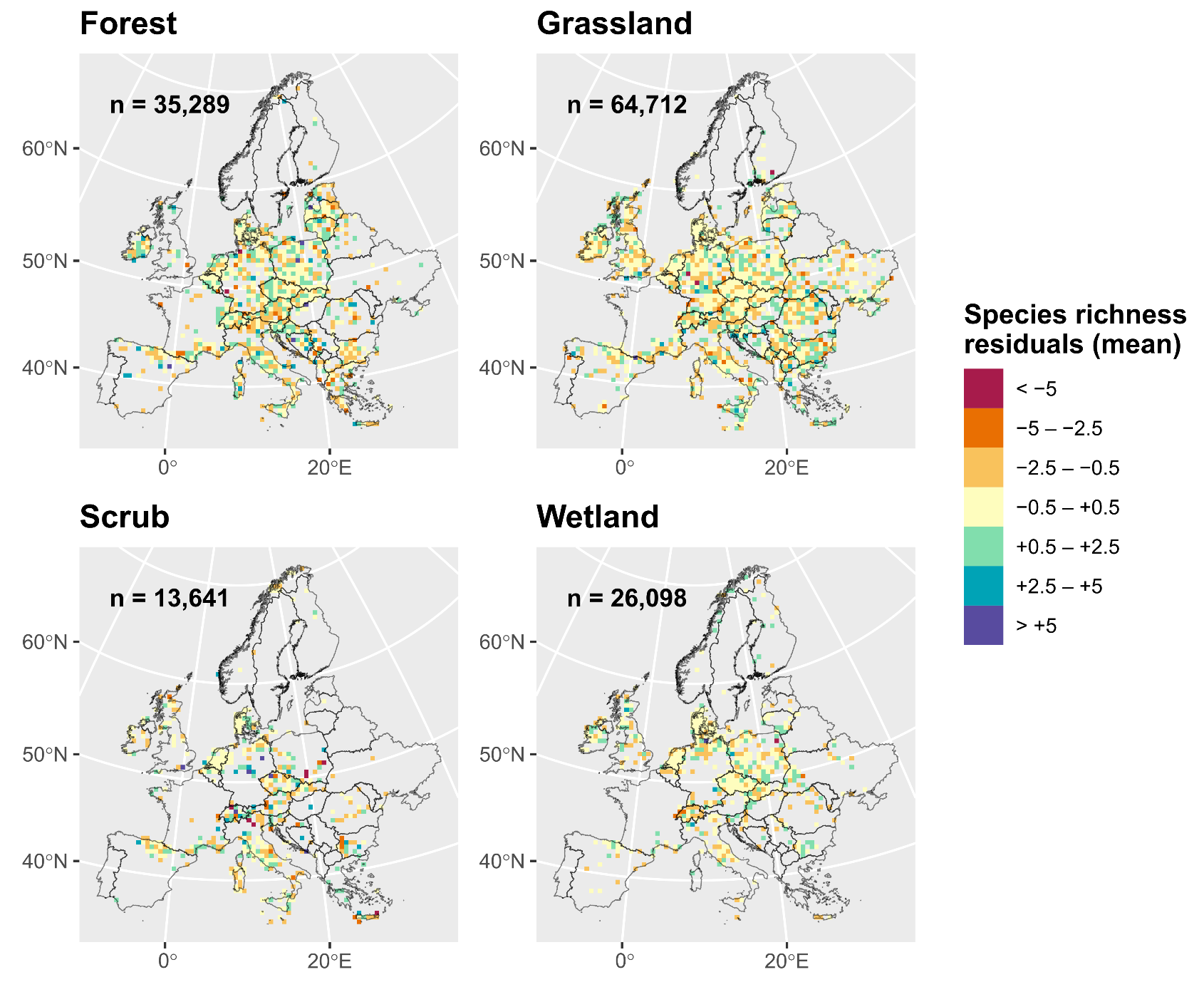
**9**: Spatial distribution of model residuals. The map shows the distribution of species richness residuals (observed - predicted) across Europe from the Random Forests model estimated over the testing data. Residuals were averaged within 50 km x 50 km grid cells for each habitat type. Only grid cells with at least five plots are included. The number of plots (n) is indicated within each panel. The lack of distinct geographic patterns in the residuals suggests that the model performed similarly in predicting species richness across different regions.

**Figure S10**: Interaction statistic H^2^ describing variation explained by the interactions of terms included in Random Forests. Overall, 71% of the total variation explained by the model was attributable to interactions (H^2^ statistic = 0.71, not shown in the figure). Left panel: overall interaction strength per feature H^2^_j_ (= proportion of prediction variability explained by interactions on predictor *j*). Right panel: pairwise interaction strength H^2^_jk_ (=proportion of joint effect variability of predictors *j* and *k*
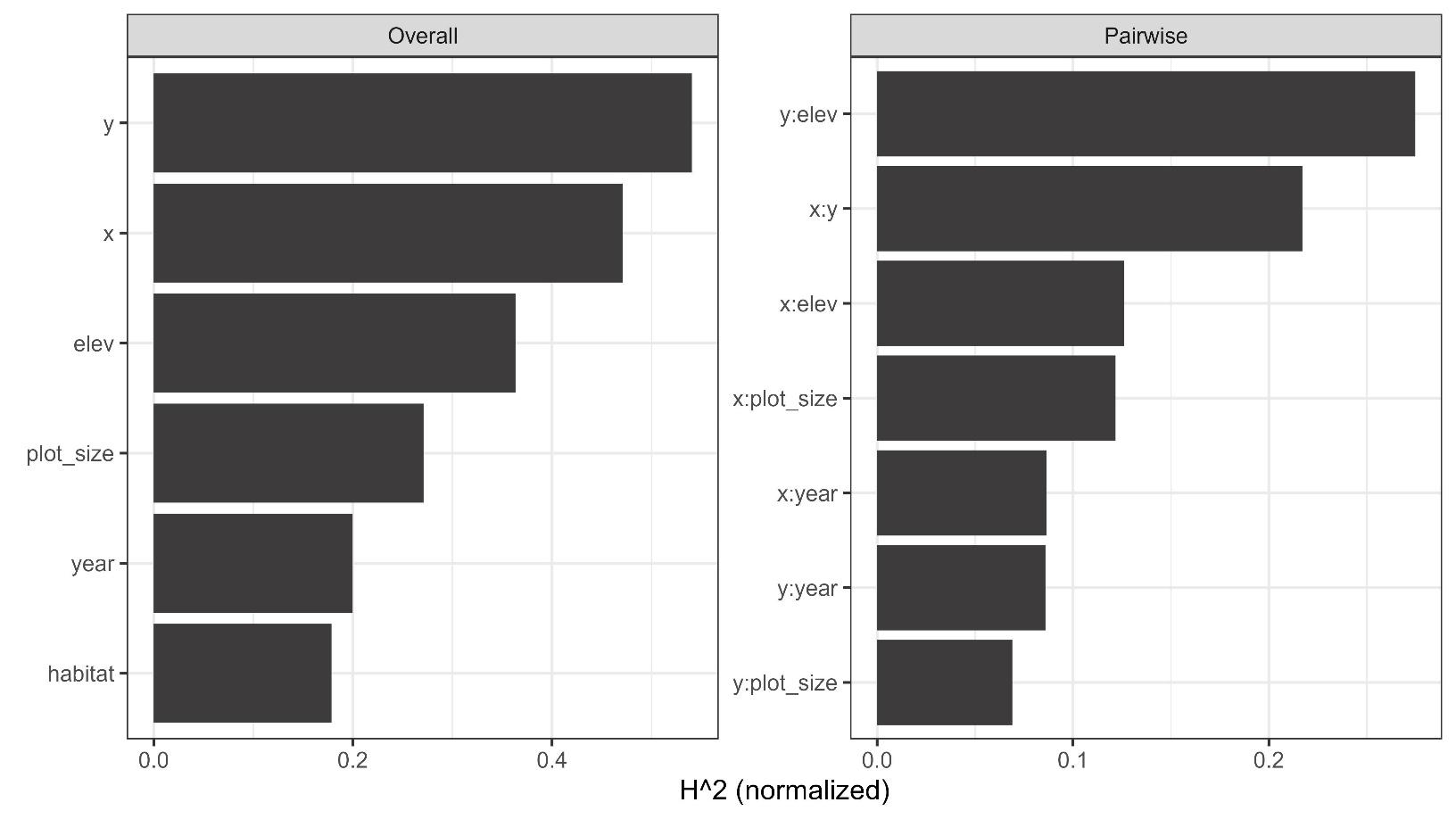
 coming from their pairwise interaction); only the top seven interactions are shown here.

**Figure S11**: Partial dependence plots and variable importance (bottom-right panel) of
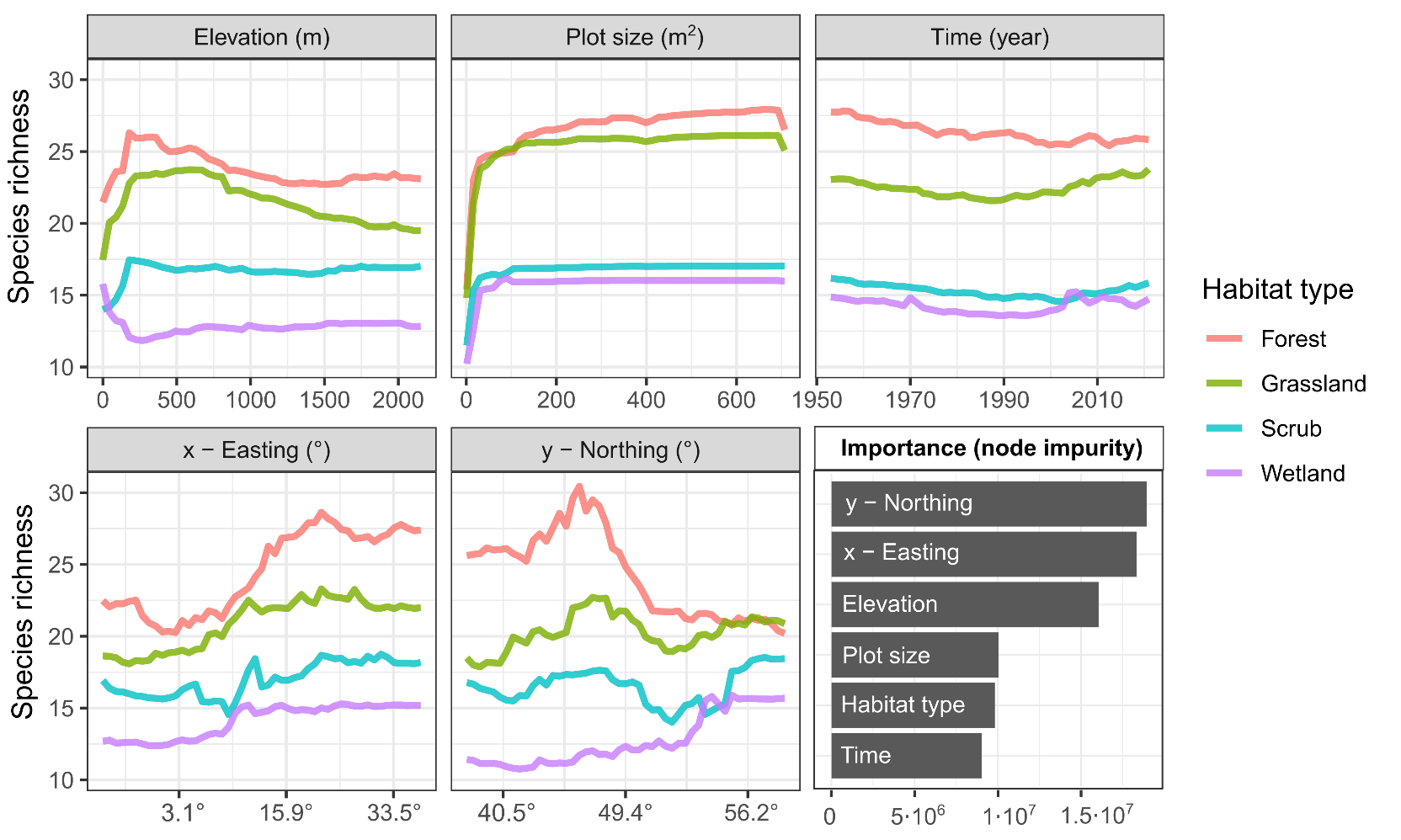
predictors in the Random Forests model used for species richness interpolation. Partial dependence plots are grouped by habitat type. Easting (longitude) and northing (latitude) are originally expressed in meters (m) (the x-axis scale is transformed to decimal degrees).

**Table S3**: Results of model validation trained on a static version of the ReSurveyEurope data only. The model was fitted 100 times, with individual plots randomly selected for each time series from the ReSurveyEurope data in each iteration. The table reports the resulting mean and standard deviation (SD) values of various evaluation metrics (root mean square error, *RMSE*; coefficient of determination using squared correlation, R^2^; Pearson correlation of observed versus predicted values, *cor*) obtained from three testing datasets. See ‘Materials and Methods’ and Figure S12 for additional details.

|  | **RMSE** | **R^2^** | **cor** |
| --- | --- | --- | --- |
| **Static *S*  (20% testing)** | 7.40  (SD: 0.1160) | 0.639  (SD: 0.0096) | 0.799 (SD: 0.0060) |
| **Static *S* (ReSurveyEU)** | 5.99  (SD: 0.0244) | 0.737 (SD: 0.0021) | 0.858  (SD: 0.0012) |
| **ΔS** **(ReSurveyEU)** | 0.386  (SD: 0.0018) | 0.415  (SD: 0.0055) | 0.645 (SD: 0.0042) |

**Figure S12**: Validation tests for the temporal interpolation of species richness dynamics. The panel matrix reports evaluation results of observed (x-axis) vs. predicted (y-axis) species richness (static S) or its derived change (ΔS). Each panel includes evaluation metrics (root mean square error, *RMSE*; coefficient of determination using squared correlation, *rsq* (= R^2^); Pearson correlation of observed versus predicted values; *cor*) and sample size (*n*). Predictions were obtained from three models trained over different dataset combinations (columns) and tested over three different testing datasets (rows). The training data are obtained from A) ReSurveyEurope only (1^st^ column), B) EVA only (2^nd^ column), and C) a combination of both (3^rd^ column), using no repeated survey. The testing dataset included 1) the 20% data from the initial split (1^st^ row), 2) all remaining plots in ReSurveyEurope not utilized for model training (2^nd^ row), and 3) species richness changes between the initial and final plots within each resurvey time series (3^rd^
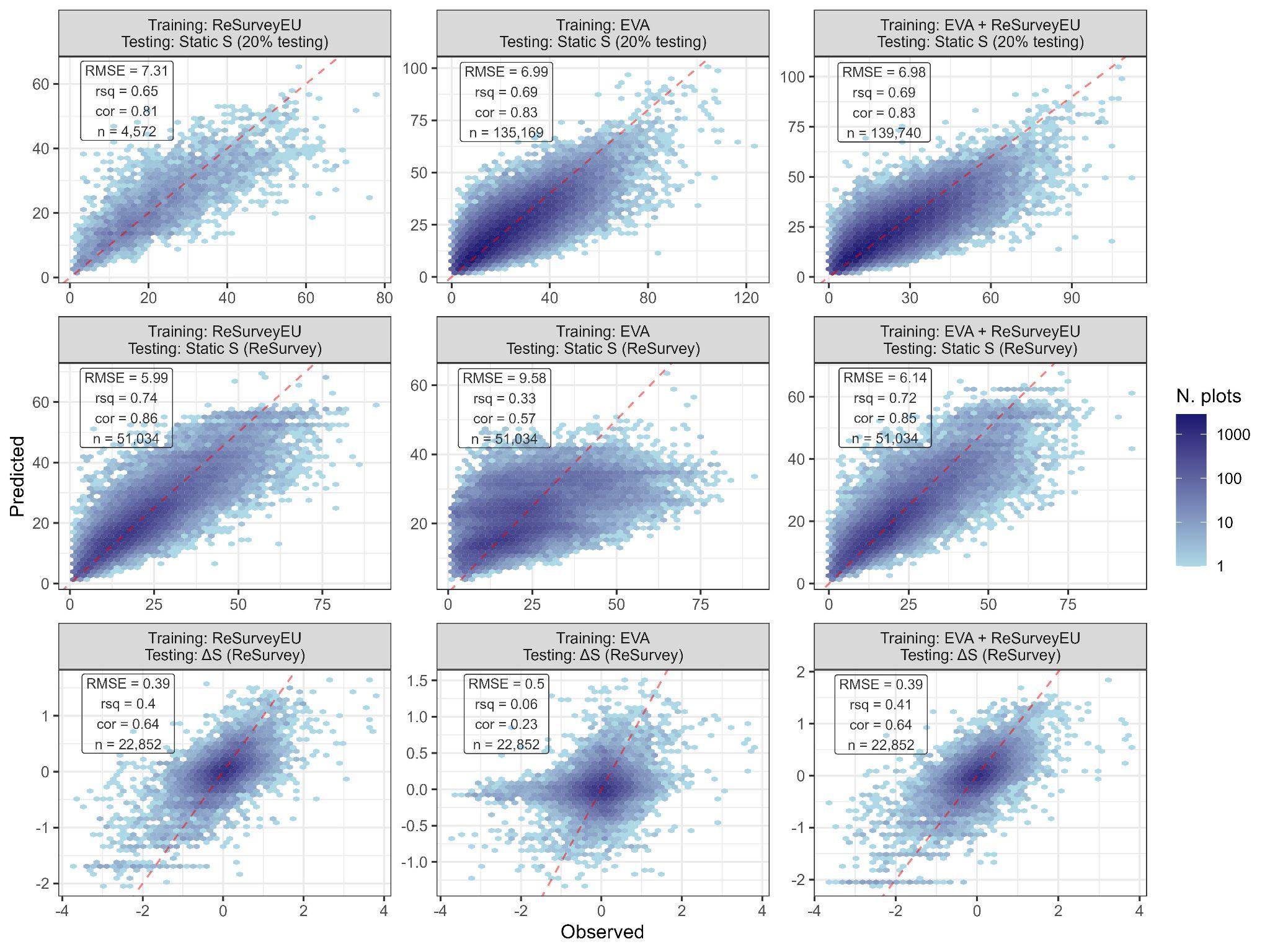
 row). See ‘Model validation’ section in ‘Materials and Methods’ for more details.


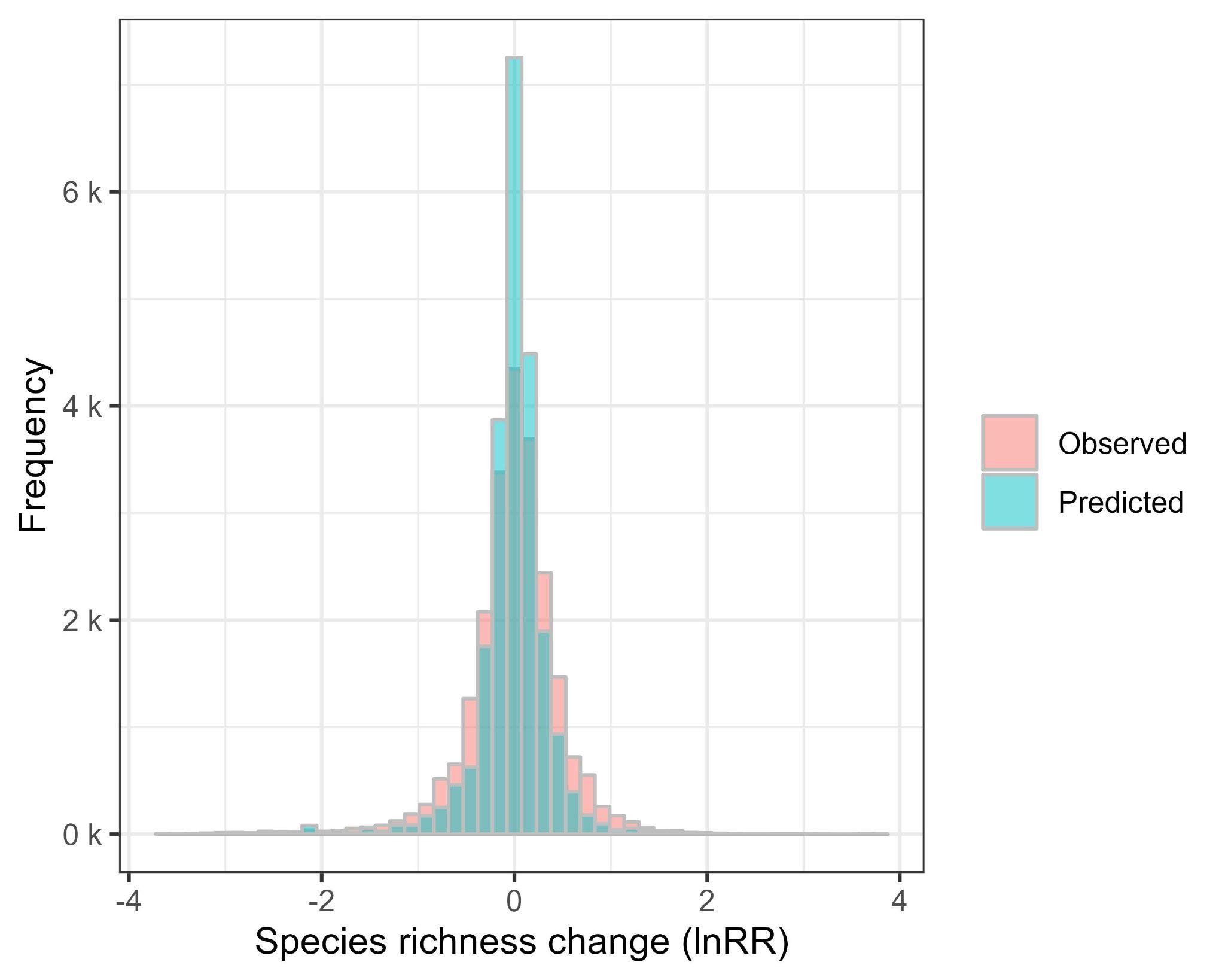
**Figure S13**: Interpolations tend to predict less strong changes than observed values. The histogram compares the distribution of observed vs. predicted species richness changes across 22,852 resurvey sites. This is calculated as a log response ratio (lnRR) between the species richness in the final plot to the one in the initial plot within each time series of ReSurveyEurope data. Predictions were obtained from a model trained on a static version of ReSurveyEurope data only (= using a single plot randomly selected for each time series). Same values are displayed in the scatter plot on the bottom-left panel of Figure S12.

**Figure S14**: Validation tests for the temporal interpolation of different training datasets (see Figure S
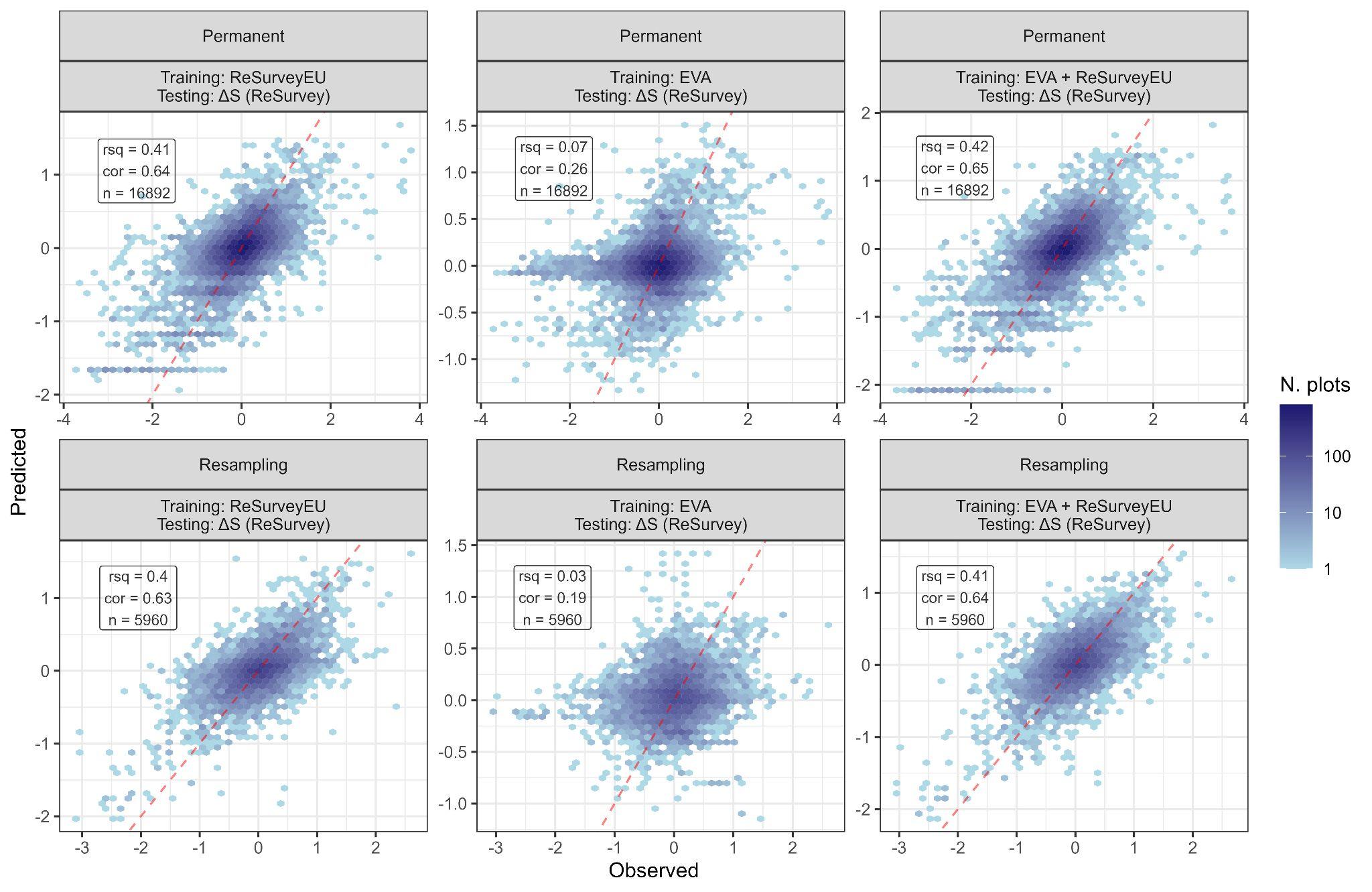
12) comparing observed vs. predicted species richness change (ΔS) over the ReSurveyEurope data. Here, separate evaluation statistics were computed based upon the type of resurvey: either permanent plots (plots resurveyed at precisely re-located sites; first-row panels) or quasi-permanent (‘Resampling’) plots lacking accurate re-location (second-row panels). No significant discrepancy in model evaluation results was detected between the two re-survey methods. Species richness change is calculated as a log-response ratio (lnRR) between the species richness in the final plot and the one in the initial plot within each time series of ReSurveyEurope.


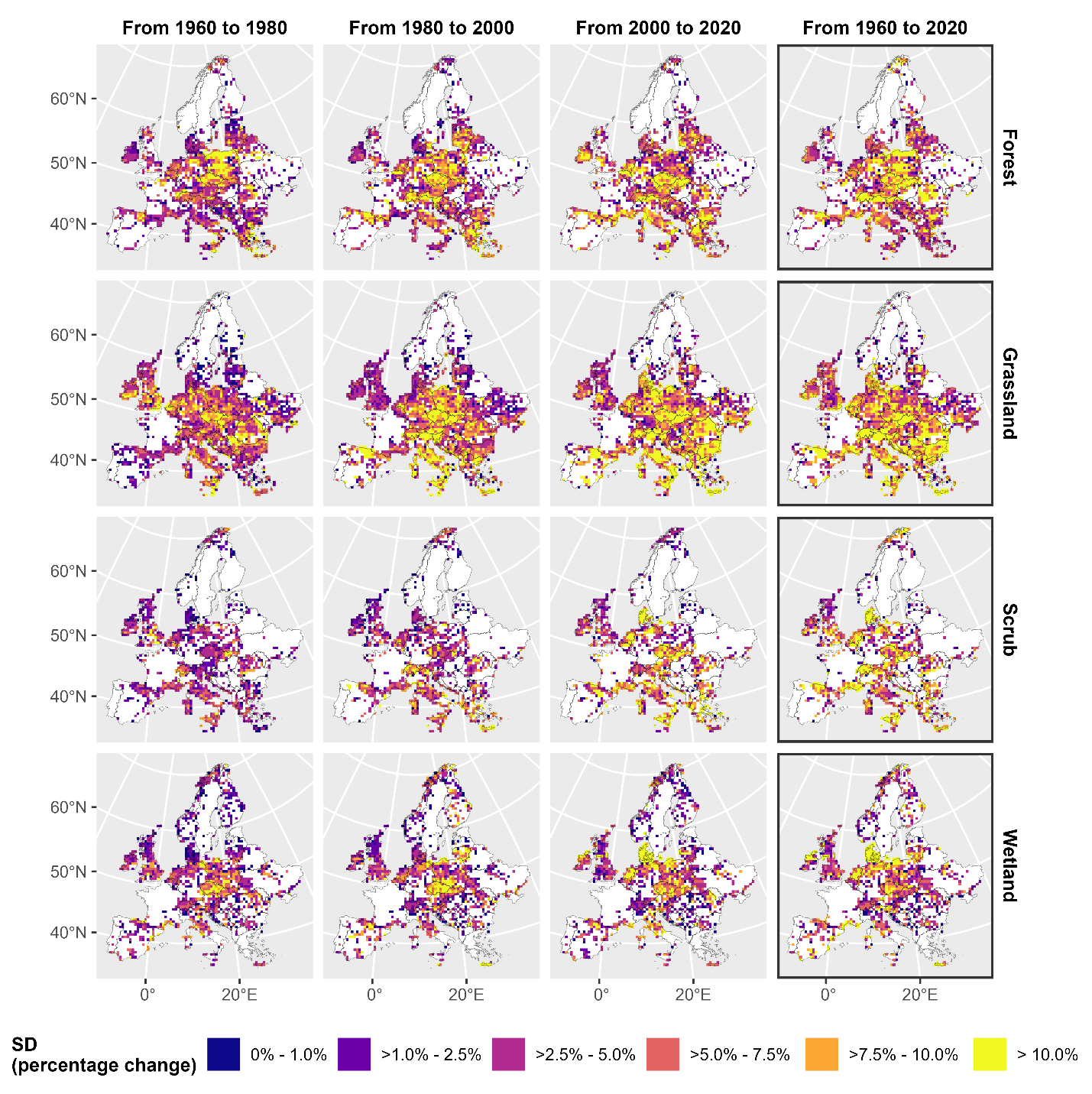


**Figure S15**: Geographical patterns of standard deviations to the mean of interpolated species richness change. The maps are based on Figure 4 of the main manuscript, displaying the standard deviations of average plot-level percentage of change of species richness between two points in time for each period, on a 50 km × 50 km grid. Like in Figure 4, only grid cells with at least five plots were displayed. To account for differences in plot sizes, we predicted species richness for each plot and year using the median plot size value for its habitat type (i.e., 300 m² for forests, 20 m² for grasslands, 64 m² for scrub, and 50 m² for wetlands).


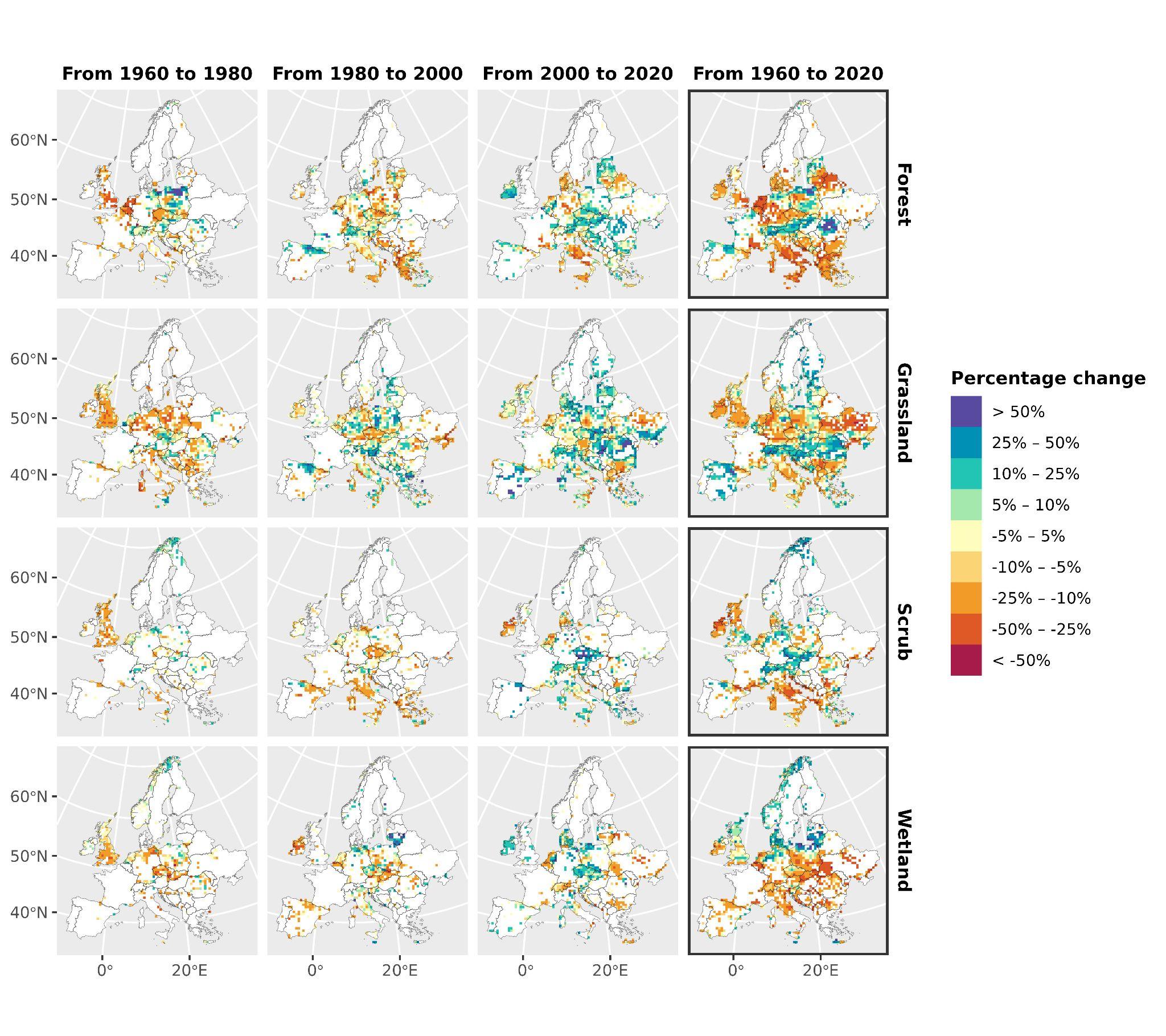
**Figure S16**: Geographical patterns of interpolated species richness change in Europe across main habitat types based on percentages of change (%). The maps are based on the average plot-level percentage of change of species richness between two points in time for each time period, on a 50 km × 50 km grid (as displayed in Figure 4 in the main text). In comparison to Figure 4, only grid cells with at least five plots sampled within each period are displayed (i.e. number of cells varies across panels depending on data availability for a given period). To account for differences in plot sizes, we predicted species richness for each plot and year using the median plot size value for its habitat type (i.e., 300 m² for forests, 20 m² for grasslands, 64 m² for scrub, and 50 m² for wetlands).


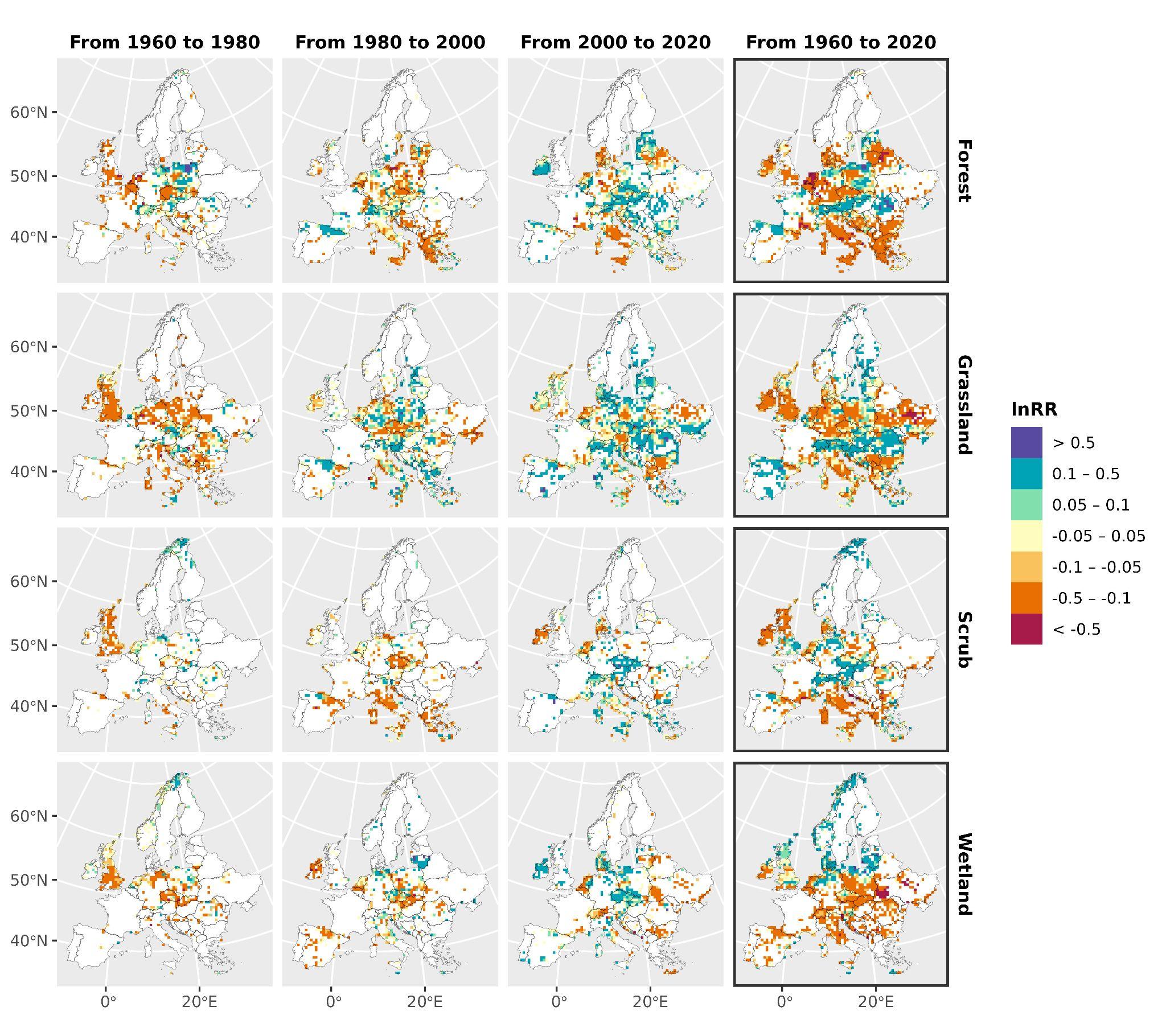
**Figure S17**: Geographical patterns of interpolated species richness change in Europe across main habitat types based on *log-response ratios* (lnRR) in each plot. The maps are based on average plot-level lnRRs of species richness between two points in time for each period, on a 50 km × 50 km grid. See Figure S11 for additional details.


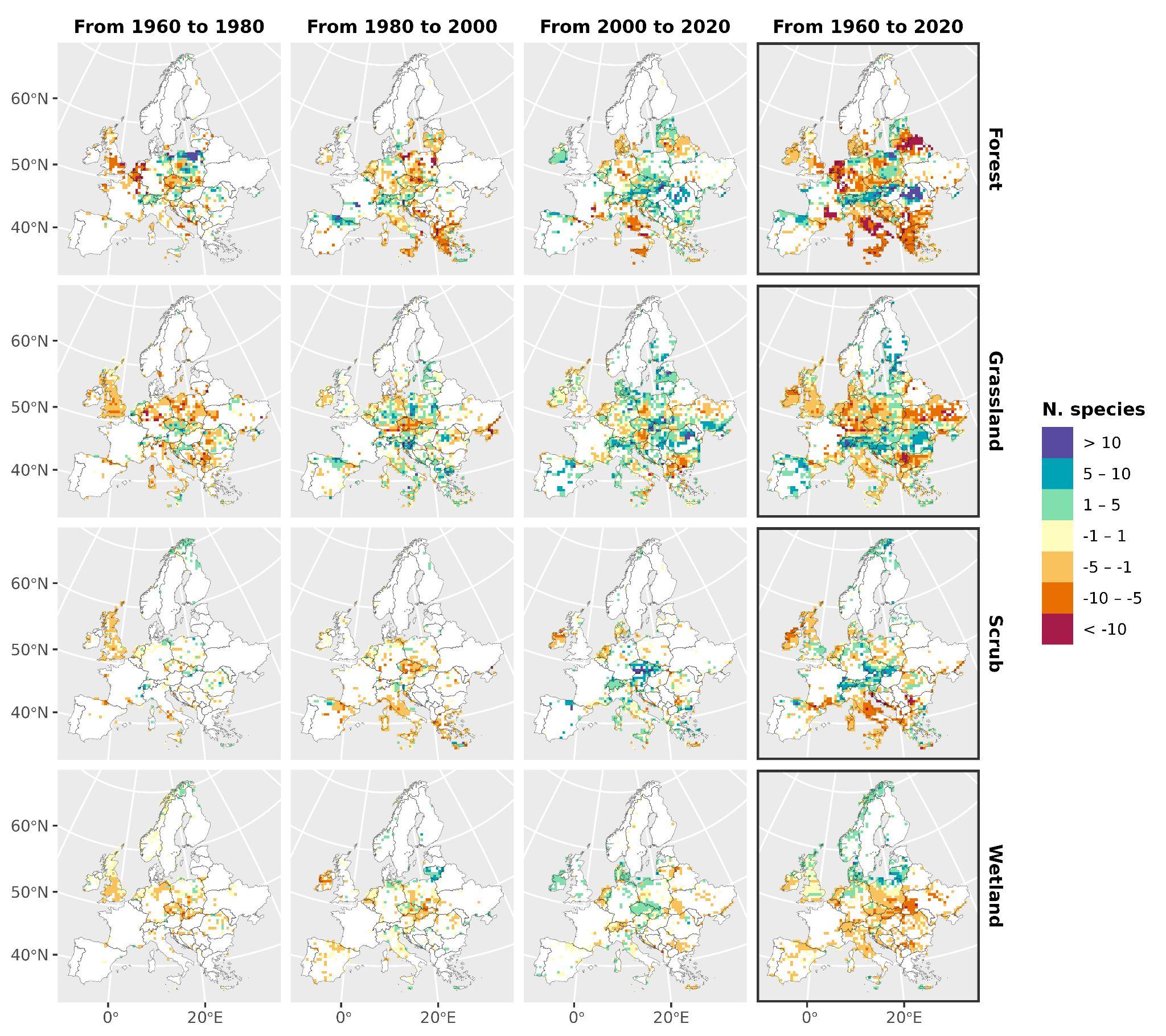
**Figure S18**: Geographical patterns of interpolated species richness change in Europe across main habitat types based on the *absolute* *number of species change* in each plot. The maps are based on the average plot-level number of species change between two points in time for each period, on a 50 km × 50 km grid. See Figure S11 for additional details.


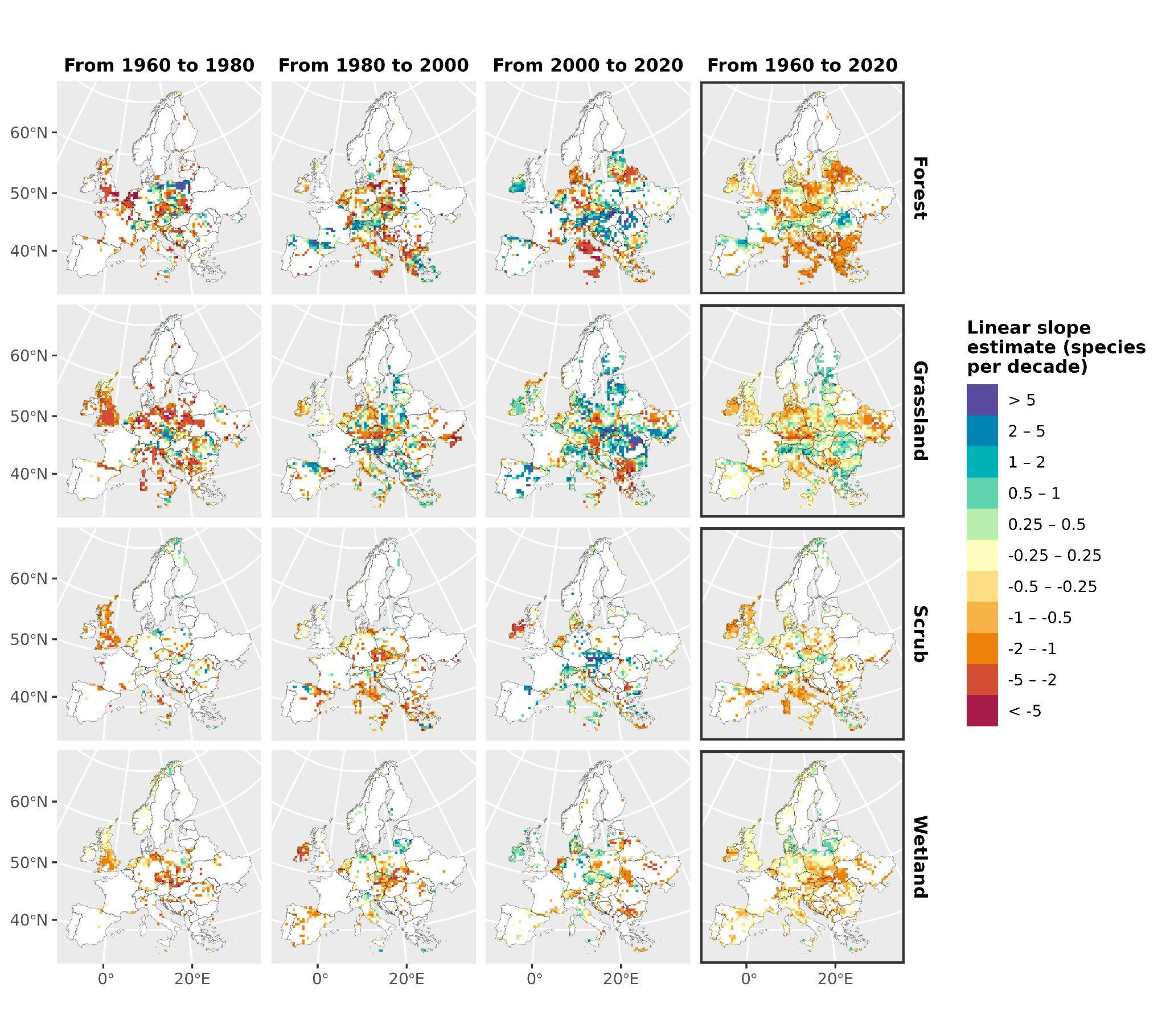
**Figure S19**: Geographical patterns of interpolated species richness change in Europe across main habitat types based on *linear slope estimates* in each plot. The maps are based on average plot-level slope estimates obtained from linear regressions of species richness against time, on a 50 km × 50 km grid. Species change per year is expressed in decades (multiplied by 10). See Figure S11 for additional details.


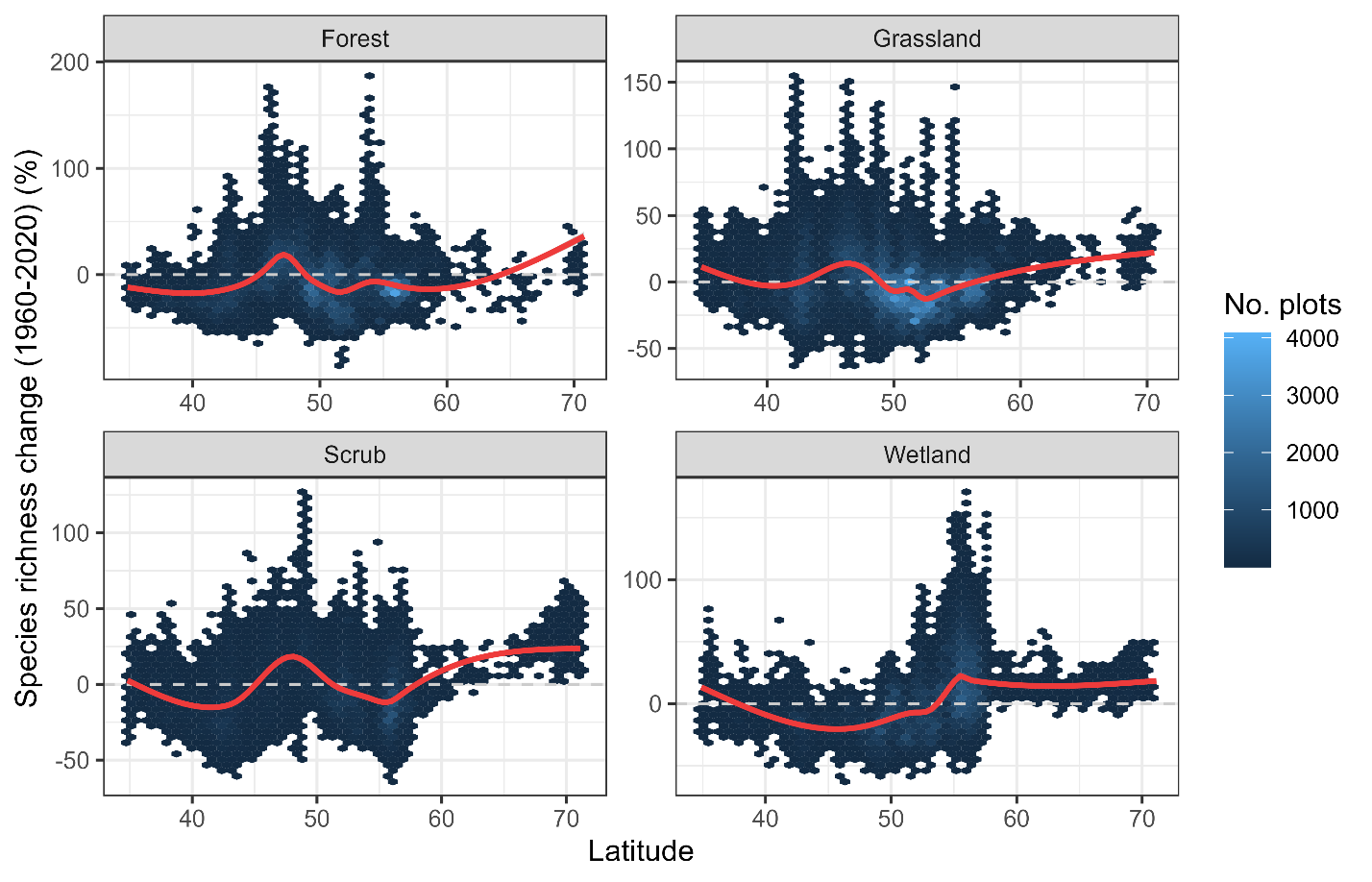


**Figure S20**: Relationship between latitude and interpolated plant species richness for the year 2020 compared to 1960 across 660,748 European vegetation plots sampled over that period. A loess smoother was used to summarize trends across various habitats.
